# Web-Based Intervention Programs for Depression: A Scoping Review and Evaluation

**DOI:** 10.2196/jmir.3147

**Published:** 2014-09-23

**Authors:** Tian Renton, Herman Tang, Naomi Ennis, Michael D Cusimano, Shree Bhalerao, Tom A Schweizer, Jane Topolovec-Vranic

**Affiliations:** ^1^Trauma and Neurosurgery ProgramSt Michael's HospitalToronto, ONCanada; ^2^Li Ka Shing Knowledge InstituteSt Michael's HospitalToronto, ONCanada; ^3^Division of NeurosurgeryUniversity of TorontoToronto, ONCanada; ^4^Medical Psychiatry ServiceSt Michael's HospitalToronto, ONCanada; ^5^Keenan Research Centre for Biomedical ScienceSt Michael's HospitalToronto, ONCanada; ^6^Department of Occupational Science and Occupational TherapyUniversity of TorontoToronto, ONCanada

**Keywords:** depression, Web-based interventions, interactive treatment, health care access, mental health, technology

## Abstract

**Background:**

Although depression is known to affect millions of people worldwide, individuals seeking aid from qualified health care professionals are faced with a number of barriers to treatment including a lack of treatment resources, limited number of qualified service providers, stigma associated with diagnosis and treatment, prolonged wait times, cost, and barriers to accessibility such as transportation and clinic locations. The delivery of depression interventions through the Internet may provide a practical solution to addressing some of these barriers.

**Objective:**

The purpose of this scoping review was to answer the following questions: (1) What Web-delivered programs are currently available that offer an interactive treatment component for depression?, (2) What are the contents, accessibility, and usability of each identified program?, and (3) What tools, supports, and research evidence are available for each identified program?

**Methods:**

Using the popular search engines Google, Yahoo, and Bing (Canadian platforms), two reviewers independently searched for interactive Web-based interventions targeting the treatment of depression. The Beacon website, an information portal for online health applications, was also consulted. For each identified program, accessibility, usability, tools, support, and research evidence were evaluated and programs were categorized as evidence-based versus non-evidence-based if they had been the subject of at least one randomized controlled trial. Programs were scored using a 28-point rating system, and evidence- versus non-evidence-based programs were compared and contrasted. Although this review included all programs meeting exclusion and inclusion criteria found using the described search method, only English language Web-delivered depression programs were awarded an evaluation score.

**Results:**

The review identified 32 programs meeting inclusion criteria. There was a great deal of variability among the programs captured in this evaluation. Many of the programs were developed for general adolescent or adult audiences, with few (n=2) focusing on special populations (eg, military personnel, older adults). Cognitive behavioral therapy was the most common therapeutic approach used in the programs described. Program interactive components included mood assessments and supplementary homework sheets such as activity planning and goal setting. Only 12 of the programs had published evidence in support of their efficacy and treatment of depressive symptoms.

**Conclusions:**

There are a number of interactive depression interventions available through the Internet. Recommendations for future programs, or the adaptation of existing programs include offering a greater selection of alternative languages, removing registration restrictions, free trial periods for programs requiring user fees, and amending programs to meet the needs of special populations (eg, those with cognitive and/or visual impairments). Furthermore, discussion of specific and relevant topics to the target audience while also enhancing overall user control would contribute to a more accessible intervention tool.

## Introduction

In October 2012, the World Health Organization estimated that 350 million people worldwide suffer from depression [1]. In Canada alone, 12.2% of the adult population has met depression diagnosis criteria within their lifetime [2]. It is expected that depression will be the world’s largest medical burden on health by 2020 [1]. Beyond its burden on society, depression is associated with worse global outcomes for the affected individual, including reduced social functioning, lower quality of life in regards to health, inability to return to work, as well as suicide [3-5].

Not all individuals face the same risk. Certain populations are more susceptible to depression relative to others. For example, it is estimated that 14%-77% of traumatic brain injury survivors suffer from depression post injury, which is a much higher than the general population [6-11]. The prevalence of depression is also moderated by gender. Research has indicated that almost double the number of women (10%) relative to men (5%) suffer from depression within their lifetime [12]. Moderated by age, young people aged 15-24 years were found more likely to experience substance abuse disorders and mental health disorders than any other age group [13].

There are a number of depression treatment options available including medication, lifestyle, and psychological interventions. Unfortunately, many therapies are unavailable to patients due to a lack of qualified service providers. In Canada, it is estimated that only 33% of individuals seeking mental health services actually receive treatment [14]. Patients also experience barriers such as prolonged wait times, cost, and accessibility barriers such as transportation and clinic locations. Within Ontario, 60% of family physicians rated accessibility to psychiatrists as fair to poor [13].

Not only are individuals hindered by accessibility issues but also by the stigma associated with a mental illness diagnosis such as depression. Only 42% of Canadians said that they would socialize with a friend suffering from a significant mental health issue [13], and 46% of Canadians believe mental illness is used as a term to excuse poor behavior [13]. Associated stigma and treatment barriers stress the need for alternative health care options. These barriers may partially explain why a recent study found that almost 49% of individuals who believe they have suffered from depression or anxiety have never sought professional health care [15].

Recent advances in computer technology offer potential alternative treatment options. The percentage of the global population that had access to the Internet nearly doubled from 18% in 2006 to 35% in 2011 [16]. As the scope of applications supported by technology and their use have increased over time, educational resources and Web-based treatment interventions have emerged to target mental health issues. At present, treatment programs are available for eating disorders [17], smoking cessation [18], obesity [19], safe sexual practices [20], autism spectrum disorders [21], substance use disorders [22], physical activity for type 2 diabetics [23], lifestyle behavior improvement [24], cannabis use [25], reducing chronic obstructive pulmonary disease risk behaviors [26], mental health in tertiary students [27], and chronic illness management [28], as well as others.

Regarding Web-based intervention treatments for depression specifically, the Canadian Network for Mood and Anxiety Treatments suggested computer-assisted cognitive behavioral therapy (CBT) programs as a second line treatment in cases “where first line treatments are not indicated or cannot be used or when first line treatments have not worked” [29]. However, programs must provide at a minimum Level 3 evidence (non-randomized controlled prospective studies or case series or high quality retrospective studies) and additional clinical support [29]. Similarly, the Improving Access to Psychological Therapies program of the UK National Institute for Health and Clinical Excellence recommends computerized CBT as a low-intensity intervention for depression [30].

Reviews of existing Web-based programs for the treatment of depression are available on the Beacon website [31], as well as systematic reviews of the effectiveness of such programs [32-34]. On the Beacon website, health care experts review, categorize, and rate Web-based programs, mobile applications, and support groups. A brief summary is provided for each site including a description of the program, details of the site, target audience, access requirements, and the extent of research evidence in support of the program. A rating is provided for each site based on the degree to which research evidence is available. Currently, 45 websites, 5 mobile apps, and 8 support groups have been identified and reviewed on the Beacon site.

We conducted a scoping review of interactive Web-based treatment programs for depression to answer the following questions: (1) What Web-based programs are currently available that offer an interactive treatment component for depression?, (2) What are the contents, accessibility, and usability of each identified program?, and (3) What tools, supports, and research evidence are available for each identified program? Unlike the systematic reviews that have been published to date, our review included publicly available Web-based programs with and without supporting research trials. In addition, we have compared and contrasted those with and without supporting evidence on predetermined evaluation criteria.

## Methods

### Search Strategy

A search for interactive programs targeting depression was conducted in April 2014 using Google, Yahoo, and Bing (Canadian versions)—popular and comprehensive search engines easily accessible to many individuals. A list of the search terms used is included in Textbox 1. A program was included in the review if it (1) targeted the treatment of depression, (2) was accessible via the Internet, (3) had an interactive treatment component (ie, was not purely educational) and required user participation (ie, homework, worksheets, mood assessment), and (4) was available in English (additional languages allowed). A program was excluded if it (1) solely provided information regarding depression (ie, psychoeducation), (2) solely targeted the treatment of mental health issues other than depression (eg, anxiety, bipolar disorder, post-traumatic stress disorder), (3) was not accessible to the public (private programs), (4) solely targeted health care professionals for training purposes, (5) offered only mood tracking applications, (6) required supplemental equipment that would not be publicly available, (7) was solely available for research purposes (ie, user must be enrolled in the study to access the program), (8) offered no treatment program, (9) could not be completed within the home or private setting (ie, must attend classes), and (10) offered online counseling only (ie, no program associated with the website).

For the purposes of this scoping review, interactive was defined as a program requiring user engagement and input (eg, mood assessments, user worksheets, and integrated program requirements for mandatory user feedback). If a program provided only reading materials on the symptoms of depression, treatments, and relapse prevention, it was classified as psychoeducational and did not meet the “interactive” inclusion criterion.

**Search terms used in the search engines Google, Bing, and Yahoo.**

1. Online depression treatment
2. CBT depression online treatment
3. Depression and CBT online programs
4. Online methods of depression treatment
5. Computerized cognitive behavior therapy programs for treatment of depression
6. Online cognitive behavior therapy programs treating depression
7. Computer based depression treatment programs
8. Internet delivered depression treatment programs
9. E-therapy for depression

Each of the terms in Textbox 1 was entered into the three identified search engines. A search log outlines the number of hits included and excluded, in addition to duplicate entries (see Multimedia Appendix 1). A search was terminated for the term when five consecutive pages of search results failed to identify any new programs. The page on which the search was terminated is included in the search log.

The Beacon website [31] was additionally investigated for programs meeting our inclusion criteria. Nine additional programs were identified from Beacon that had not been identified using our search strategy. Two independent reviewers completed the search as indicated above, resulting in only five discrepancies among the programs identified. After careful evaluation, a consensus was reached regarding included programs, with the search providing 20 programs and Beacon an additional nine programs. The remaining three programs were previously known to reviewers: After Deployment, Students Against Depression, and Dealing With Depression.

### Program Evaluation Criteria

In order to systematically evaluate each identified program, we used criteria adapted from previously published guidelines [35] to generate five categories of evaluation that were broken down into 14 subcategories. Table 1 provides a detailed description of the category of assessment, the investigation, and the evaluation focus.

**Table 1:** Categories of investigation used to evaluate each program.

Category	Investigation	Evaluation focus
Accessibility	Fee/Referral	Was there a fee or physician referral required to access the program?
	Language	Was the program available in alternative languages?
	Registration Requirement	Was personal information required to access the program? Were details provided regarding the registration process?
	Target Audience	Who was the program designed for?
Usability	Statistics	Registered users, completion rates, and attrition data (if available).
	Therapeutic Approach	What therapies/treatment approach(es) were offered?
	Mode of Delivery	How was the content delivered: size of text, audio or video offered, use of character examples, and case scenarios?
Tools	Additional Features	What additional features were available (eg, if users could monitor their progress/modules completion and mood over time)? Were email reminders and follow-up offered?
	Worksheets	Did the program offer worksheets (printable for offline use or integrated throughout program)? Indicated whether worksheets were mandatory or optional.
	Assessments	Were assessments offered within the program? Indicated whether assessments were mandatory for completion of program.
Support	Clinician Support	Did the program offer linking with a clinician (either user’s own clinician or program specific clinician) and type of linkage (eg, telephone monitoring)?
	Peer Support	Did the program offer peer support (eg, forum, personal story sharing, or blogs)?
	Crisis Links	Did the program offer crisis or emergency contacts?
Evidence	Randomized Controlled Trial	Had the program been evaluated for efficacy with at least one randomized controlled trial?

A program evaluation scoring system was also created based on the above mentioned article [35] to provide an objective numerical score for each of the captured programs. Permissions were obtained from the publishing journal to adapt the scoring system: 28 evaluation points were adapted from the 12 facets and guidelines discussed in the article, and facets were adapted into yes or no, closed-ended inquiry questions. Programs meeting the inquiry were awarded 1 point. Programs not meeting the inquiry were given 0 points or a 0^a^ in the case that the point could not be evaluated using the program of interest and/or attempts to contact program developers. Table 2 outlines the facets and guidelines of the adapted scoring system. Summative program scores (absolute and relative [%]) are listed in Multimedia Appendices 2 and 3. A detailed breakdown of the points awarded per program is provided in Multimedia Appendix 3.

Two reviewers independently examined each program and scored them using the evaluation criteria outlined in Table 2. The reviewers achieved initial agreement on 782 of the 812 (96.3%) evaluation points (28 possible points across 29 programs). The reviewers then met and resolved the discrepancies through further program investigation or consulting developer/reviewer email communications until 100% agreement was achieved.

An identified program was categorized as an evidence-based program (EBP) if it was the intervention in at least one published randomized controlled trial (RCT). All other programs were categorized as non-evidence-based programs (NBP).

**Table 2:** Facets and adapted evaluation criteria.

Facet # and description	Adapted evaluation description and #
1. Focus and target population	1. Were the primary focus/ goals/ objectives of the intervention stated?
	2. Was an initial assessment conducted for program/user suitability purposes?
	3. Was the target audience or age group defined?
2. Authorship details	4. Were the names and credentials of authors present?
	5. Was the ownership or developer name provided?
	6. Were links to the developer website provided?
	7. Was date of program/site update provided?
	8. Was country of origin stated?
3. Model of change	9. Was the model of change (ie, type of therapy utilized) defined/stated?
4. Type and dose of intervention	10. Were the number of modules or time to complete each module stated?
	11. Was the program structured/guided (ie, modules to be completed in a restricted and specific order =1) or unstructured/unguided (ie, modules could be accessed freely =0)?
	12. Was the intervention tailored to the user or was it generic for all users?
	13. Did users receive feedback?
	14. Could users track their progress throughout the program?
	15. Were the assessments validated/reliable?
5. Ethical issues	16. Were the risks of the program stated/benefits of program were stated?
	17. Were safe guards provided (ie, crisis links /telephone hotline numbers provided)?
	18. Was a unique user name or password provided to users?
	19. Was the site secure?
	20. Were the rights and use of user personal information provided?
6. Professional support	21. Was there a statement of professional support (ie, therapist integrated into the intervention)?
	22. For programs utilizing therapist support: were the credentials of the therapist provided?
7. Other support	23. Was support provided from additional sources (ie, peer discussion forums or blogs)?
	24. Was this type of support monitored by an overseeing authority?
8. Program interactivity	25. Was the interactivity of the program described and accurate (ie, how much time needing to be spent on module/homework assignments)?
9. Multimedia channel of delivery	26. Did the program offer a multimedia content delivery (ie, a combination use of text, video, graphics, and audio formats)?
10. Degree of synchronicity	This evaluation point was combined with point 13.
11. Audience reach	This evaluation point is available in Multimedia Appendix 2, under the heading “Target Audience”
12. Program evaluation	27. Was evidence for the program provided to the user (ie, attrition data/ success rate/ completion rate/ # of users in the program/ testimonials)?
	28. Was a randomized controlled trial completed for the program?

## Results

### Summary

We identified 27 websites collectively offering 32 programs in accordance with inclusion and exclusion criteria (see Multimedia Appendix 2 for identified program names). Two websites offered multiple programs targeting the treatment of depression: eCentre Clinic (The Mood Mechanic Course, The Wellbeing Course, The Wellbeing Plus Course, and The UniWellbeing Course), and This Way Up (Clinical Course, Worry and Sadness Self-Help Course, and Schools Course).

Three programs meeting inclusion criteria were not found using the searches conducted but were previously known to authors: After Deployment, Dealing With Depression, and Students Against Depression. Three programs (Interapy, Kleur Je Leven, and Internetpsykiatri) included in Tables 3-5 and Multimedia Appendix 2 were not included in the evaluation scoring log (Multimedia Appendix 3) due to language barrier restrictions. In sum, 32 programs met inclusion criteria and were included in this review for evaluation. Tables 3-5and Multimedia Appendices 2 and 3 are organized alphabetically and also by whether the program was evidence-based or not (EBP vs NBP). The first 12 programs included in Tables 3-5 and Multimedia Appendices 2 and 3 were evidence-based (see Multimedia Appendix 4 for citations) with the remaining 20 programs having no identified evaluative RCTs. See Multimedia Appendix 5 for program screenshots.

### Accessibility

#### Country of Origin

**Figure 1:**
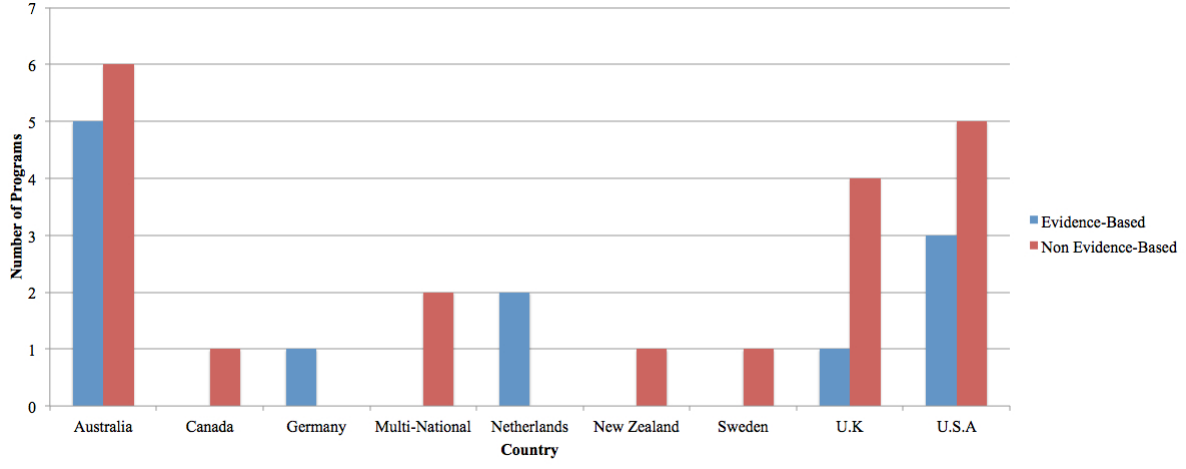
Program development by country of origin.

A summary of the countries where programs were developed is provided in Figure 1. The countries of origin for the EBP included Australia, Germany, Netherlands, the United Kingdom, and the United States. The countries of origin for the NBP were Australia, Canada, multinational collaborations, New Zealand, Sweden, the United Kingdom, and the United States.

#### Language

Four of the EBP were offered in alternative languages (Deprexis: Swedish and German; eCentre Clinic: Wellbeing Course: Arabic; MoodGYM: Simplified Chinese, Dutch, and Norwegian; and MoodHelper: Spanish). Two programs were available only in Dutch (Interapy and Kleur je Leven). Only one NBP was offered in another language and was not available in English (Internetpsykiatri: Swedish only). The remaining 19 programs were offered exclusively in English.

#### Fees or Referrals and Registration

**Figure 2:**
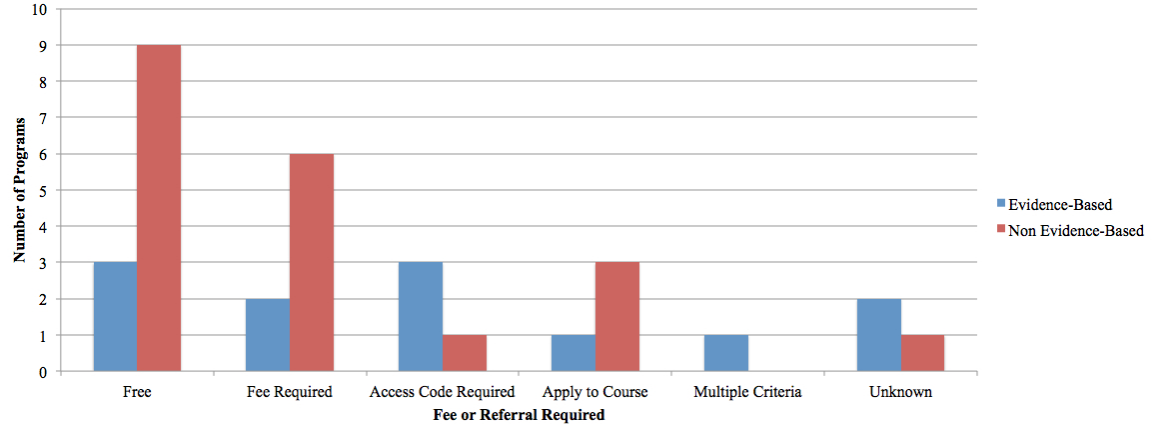
Fees and referrals required for program access.

**Figure 3:**
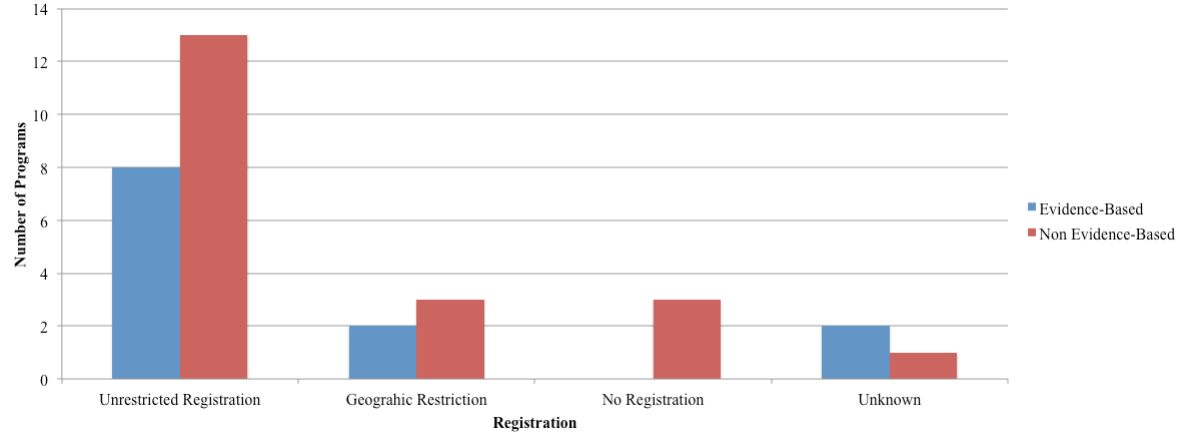
Registration required for program access.

The registration processes and fee structures varied by program for both the EBP and NBP and included those that were freely available (n=12), those with a fee required (n=8), those with an access code required (n=4), those for which an application to the course was required (n=4), those with multiple registration criteria (n=1), and those with unknown registration criteria (n=3). See Figures 2 and 3.

Eight programs had an associated fee ranging from AUD $55 (This Way Up, Clinic Course) for five/six lessons to US $400 for eight sessions (The National Stress Clinic). Most programs offered a free trial period, allowing users to interact with the program prior to paying the fee.

Most programs (n=21) required the input of personal information to access course material. Registration was restricted to certain countries for five programs (eCentre Clinic: The Wellbeing Course,The Mood Mechanic Course, The Wellbeing Plus Course, and The UniWellbeing Course, restricted to Australia; MoodHelper, restricted to the United States). Registration was not required for three programs, and registration requirements were unknown for three programs.

The programs that did not mandate registration allowed users to access program content without entering any personal information. However, users could not track their progress through these programs without registration. Programs with mandatory registration required users to input basic personal information before gaining access to program content, allowing for sessional data storage. For example, registration allowed users to create personal profiles through which mood assessments, worksheets, and module progression were recorded.

#### Target Audience

**Figure 4:**
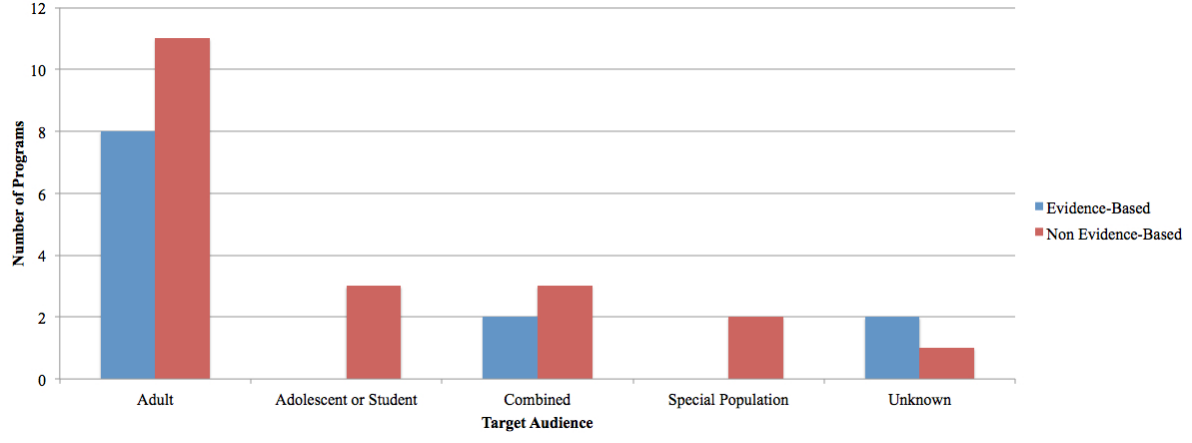
Target audience(s) of the programs.

The identified programs predominantly targeted adult audiences (n=19), with a few programs developed for adolescents or students (n=3), combined populations (ie, adolescent and adult population; n=5), a specified special population (n=2), or unknown audiences (n=3) (see Figure 4).

The majority of programs contained content that was specific to the adult end-user. Sessions varied by program and offered an array of materials targeting adult concerns surrounding depression. Topics included but were not limited to problem solving, goal setting and planning, tackling financial issues, workplace stress, in addition to challenging negative thoughts. Programs targeting adolescents were mainly focused on academic life and the stressors associated with the learning environment. Topics included time management, stress associated with exams, relationships, confidence and self-esteem, as well as other social issues faced by youth.

Two NBP were developed for specific target populations: After Deployment (discharged military personnel) and eCentre Clinic Wellbeing Plus Course (older adults). Sessions covered many issues faced by these individuals including post-traumatic stress syndrome, insomnia, depression, and anxiety.

### Evaluation Score

Evaluation scores for EBP ranged from 68%-92%, with an average score of 80%. Evaluation scores for NBP ranged from 39%-92%, with an average score of 73%. A detailed breakdown of scores per program is provided in Multimedia Appendix 3.

### Usability

#### Program Statistics and Attrition

A number of programs (n=17) provided user statistics and/or user reviews. Varying by program, statistics aimed to demonstrate overall program efficacy and effectiveness using program completion rates, number of registered users, number of users completing the course, and reductions in validated measure scores from pre- to post-intervention. See Table 3 for usability results.

**Table 3:** Usability of the programs evaluated.

Ref. #	Statistics available (# of users registered, number of unique visitors, attrition data, stated completion rate)	Therapeutic orientation/Intervention offered^a^	Content delivery^b^
1	89% of users rated the program as useful, 70% of users (mild to moderate depression) who completed the program required no further treatment	CBT	ANI/TXT++/VID
2	—	PE/CBT	AUD/TXT/VID/ANI
3	7000 patients currently enrolled in trials	CBT/PP	AUD/TXT++
4	Have offered free treatment to more than 4000 Australians	CBT/IPT	TXT++
5	—	CBT	ANI/TXT++
6	10,804 new registrations, 71,113 unique visitors, 9,199,943 Internet hits	CBT/IPT/PS/RX/PA	TXT++/VID
7	—	UNK	UNK
8	—	UNK	UNK
9	750,000 registered users	CBT	TXT++/ANI
10	Over 2500 users registered	CBT	TXT+/ANI
11	75% of users complete the course and require no further treatment, 6000 patients enrolled and 2400 clinicians as of Dec. 2012	CBT	ANI/TXT+
12	—	CBT	ANI/TXT+
13	—	UNK	AUD/TXT+/VID
14	54% average reduction in Patient Health Questionnaire (PHQ)-9 Score	MD/CBT	AUD/TXT+/VID
15	Reported decreases in Beck Depression Index scores and reportedly 60% of individuals who finish program will be “cured” of their depression/ testimonials	CBT	TXT++/ANI
16	—	CBT	VID/TXT++
17	—	UNK	AUD/TXT++/VID
18	Have offered free treatment to more than 4000 Australians	CBT/IPT	TXT++
19	Have offered free treatment to more than 4000 Australians	CBT/IPT	TXT++
20	Have offered free treatment to more than 4000 Australians	CBT/IPT	TXT++
21	—	UNK	UNK
22	222,078 registered users	CBT	ANI/TXT++
23	—	CBT/PP	VID/TXT++/AUD
24	600,000 users registered, 437,507 unique visitors, 79,607,184 Internet hits.	CBT/IPT	TXT++/ANI
25	—	CBT/IPT/PS/PP	TXT++
26	—	CBT	TXT+++
27	Testimonials and user reviews	CBT	UNK
28	User reviews	CBT	TXT++/ VID
29	—	CBT	AUD/TXT+++
30	User reviews	CBT	TXT++
31	—	UNK	VID (subtitles avail.)/TXT+++/AUD
32	—	CBT	TXT+/ANI

^a^MD:mindfulness, PA: physical activity, PE: psychoeducation, PP: positive psychology, PS: problem solving, RX: relaxation, UNK: unknown.

^b^ANI: animations/graphics, AUD: audio files, TXT: text based (+=small text blocks, ++=medium text blocks, +++=large text blocks), VID: video files.

#### Therapeutic Approach

**Figure 5:**
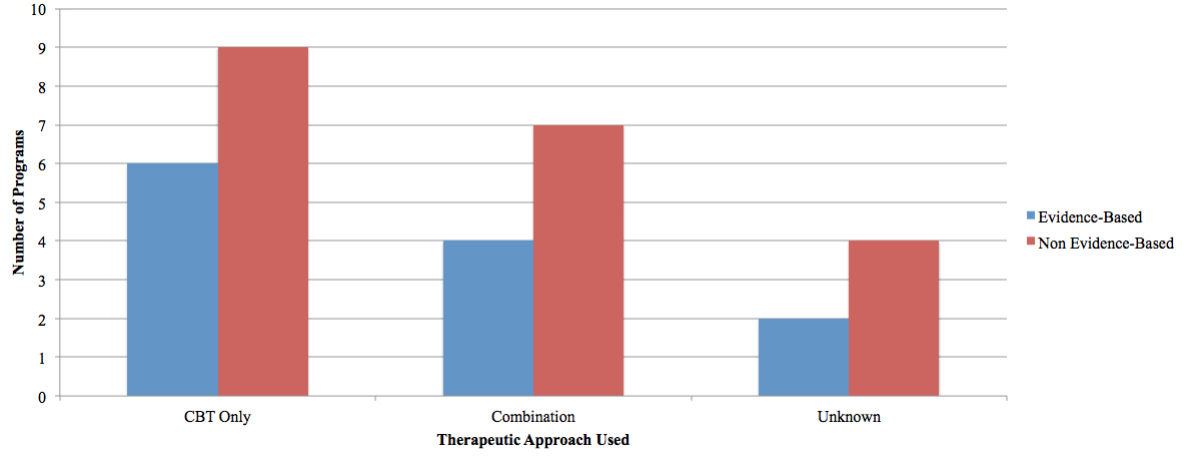
Therapeutic approach offered in programs evaluated.

Programs delivered their interventions through various therapeutic techniques. The majority of the EBP (n=6) delivered CBT-focused treatments, four offered integrated therapies (eg, CBT, Intrapersonal Therapy [IPT], psychoeducation, relaxation therapy, problem solving, and physical activity), and two did not define their therapeutic approach. Nine NBP provided CBT-based materials, seven offered combination therapy models, and four NBP were categorized as unknown as they did not define their therapeutic approach. See Figure 5.

#### Mode of Delivery

**Figure 6:**
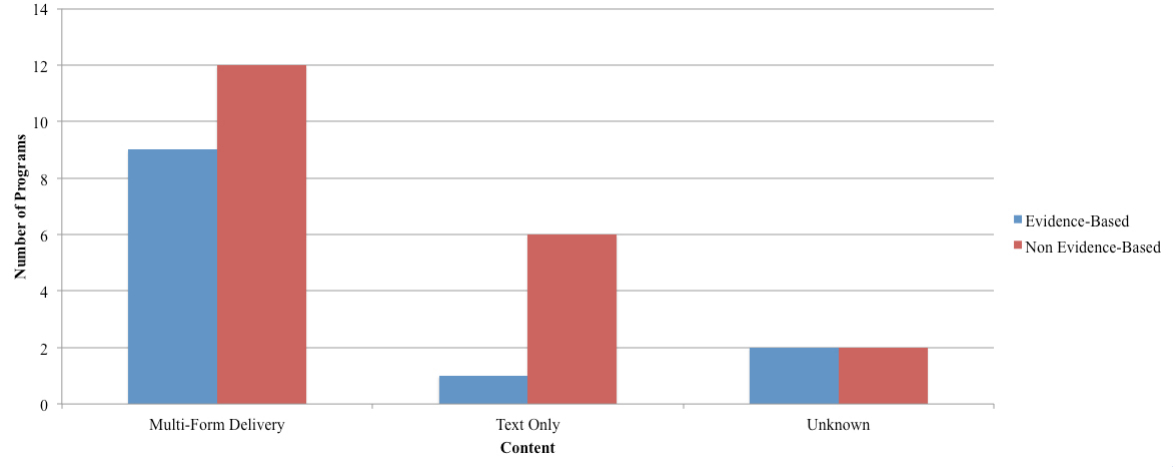
Method of content delivery.

Treatment interventions were delivered in a number of ways for both EBP and NBP. Nine EBP contained a multimodal format (ie, combination of text, video, and audio) with only one delivered in a text-only format. Two EBP were unknown (could not be evaluated). The majority of the NBP (n=12) were offered in a multimodal format. Six NBP were offered in a text-only format, and two programs were categorized as unknown. See Figure 6.

#### Quality Assessment

Table 4 provides information regarding the available tools within each program such as worksheets, assessments, and additional features (eg, email reminders, calendars, journal functions). Table 5 provides information regarding program supports from peers, from the clinician, and available crisis links.

**Table 4:** Tools associated with the programs evaluated.

Ref. #	Additional features^a^	Worksheets^b^	Assessments^c^
1	PT	W	UNK
2	PTP	W	A[V]
3	RE/MT/PTP/PT/F	W	A[V]
4	PT/F	W	A[V]
5	PT/F/MT	W	A
6	PT/MT/F	W	A[V]
7	UNK	UNK	UNK
8	UNK	UNK	UNK
9	PT/MT/F	W	A[V]
10	MT/PT/F	W	A
11	CA/PT/F	W	A[V]
12	CA/PT/F	W	A
13	PT/F/MT	W	A[V]
14	JOUR/PT/F/MT	W	A[V]
15	PTP/F/PT	UNK	A[V]
16	PT/F	W	A[V]
17	—	—	—
18	PT/F	W	A[V]
19	PT/F	W	A[V]
20	PT/F	W	A[V]
21	UNK	UNK	UNK
22	MT/PT/PTP/F	W	A[V]
23	PT/F	W	A
24	MT/PT/JOUR/PTP/F	W	A
25	PTP/F	W	A
26	PT/F/RE	W	A
27	PT/F/MT	W	A
28	MT/CA/PT/F/JOUR	—	A[V]
29	—	W	—
30	MT/PT/F	W	A[V]
31	PT/CA/F	W	A[V]
32	PT/F	W	A[V]

^a^CA: calendar application, JOUR: journal application, MT: mood tracking, F: feedback provided, PT: progress tracking, PTP: personalized treatment plan, RE: reminder emails.

^b^UNK, unknown, W: worksheets available.

^c^A: assessments available, A[V]: assessment validated.

**Table 5:** Support associated with the programs evaluated.

Ref. #	Peer support^a^	Clinician support^b^	Crisis links^c^
1	—	TC	UNK
2	—	—	UNK
3	—	TC	EC
4	—	TC	EC
5	—	—	EC
6	UNK	TC/—	UNK
7	UNK	UNK	UNK
8	UNK	UNK	UNK
9	—	—	EC
10	—	TC	EC
11	—	TC	EC
12	—	—	EC
13	PSF	—	EC
14	UNK	TC	—
15	—	—	—
16	—	—	EC
17	—	—	—
18	—	TC	EC
19	—	TC	EC
20	—	TC	EC
21	UNK	UNK	UNK
22	PSF	TC	EC
23	PSF	—	—
24	—	—	EC
25	PSF	—	—
26	PSF	TC	EC
27	PSF	TC	EC
28	—	—	EC
29	PSF	—	EC
30	PSF	—	—
31	—	TC	EC
32	—	—	EC

^a^PSF: peer support forum.

^b^TC: therapist contact via telephone/email and/or therapist linking to program, UNK: unknown.

^c^EC: emergency contact information provided.

### Tools

#### Additional Features

**Figure 7:**
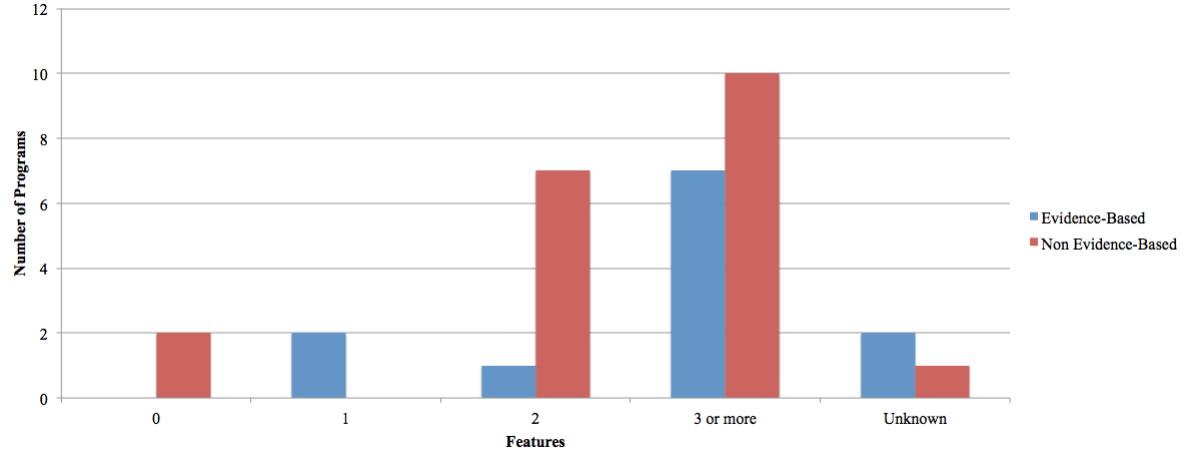
Additional features offered to program users.

Many programs included additional features such as email reminders, calendar applications, journal space, progress tracking reports as well as mood tracking. EBP and NBP both offered a number of support tool options, with most programs offering multiple features (EBP=7, NBP=10). See Figure 7.

#### Worksheets

Many programs provided lesson reinforcement activities and worksheets (EBP: n=10, NBP: n=16). Worksheets covered topics discussed in current or previous modules and encouraged user engagement. Many worksheets incorporated activity planning, goal setting, problem solving, and thought evaluation. Self-reflection activities further identified troublesome areas and encouraged corrective action.

#### Assessments

Most programs incorporated mood or depression assessment tools (EBP: n=8, NBP: n=17). Assessments were delivered prior to user registration, integrated into module content and/or independent of session programming. Programs administering assessments provided feedback and results immediately upon user completion. When evaluating some programs (EBP: n=3, NBP: n=1), it was unclear whether assessments were administered. In total, 17 programs used validated measures: Patient Health Questionnaire (PHQ)-9, Beck Depression Inventory (BDI), and Center for Epidemiologic Studies Depression Scale (CES-D).

### Support

#### From Peers

**Figure 8:**
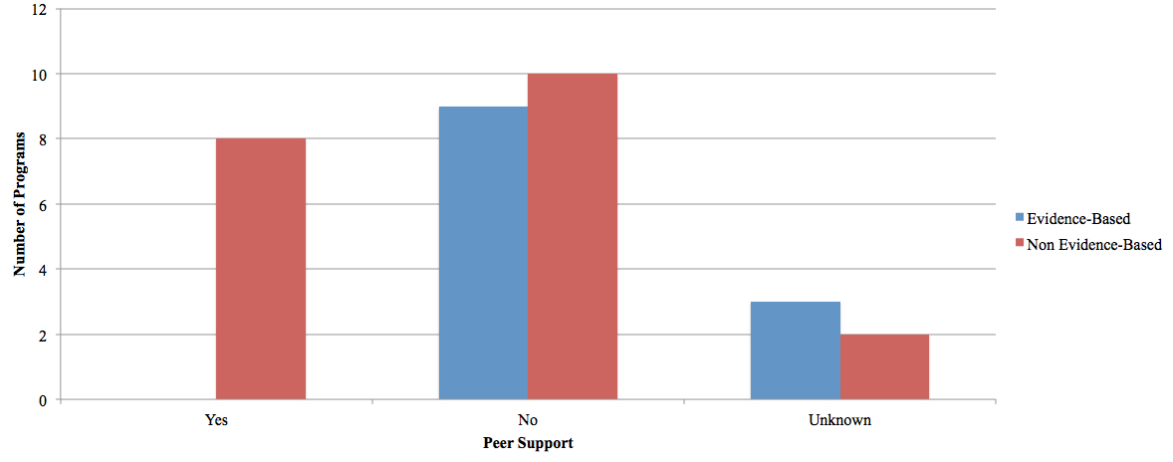
Support available from peers.

None of the EBP offered a peer-support forum (three programs were categorized as unknown). The majority (n=11) of NBP did not include a peer-support forum (one program was categorized as unknown). Only eight NBP were found to have this service available to its users. See Figure 8.

#### From the Clinician

**Figure 9:**
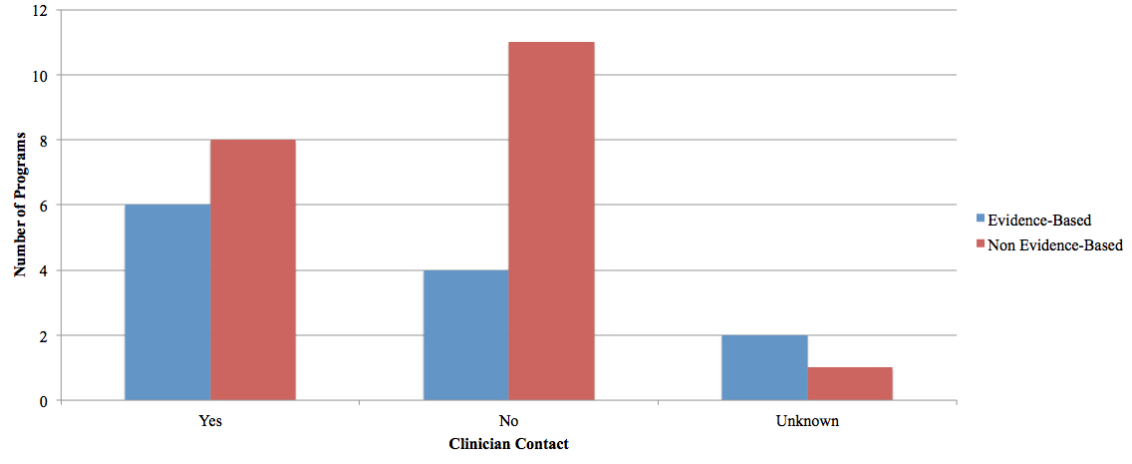
Support available from clinicians.

Six EBP offered clinician support, four offered no clinician support, and two programs were unknown. Eight NBP offered clinician support, 11 programs did not offer clinician support, and one program was not evaluated. See Figure 9.

#### Crisis Links

**Figure 10:**
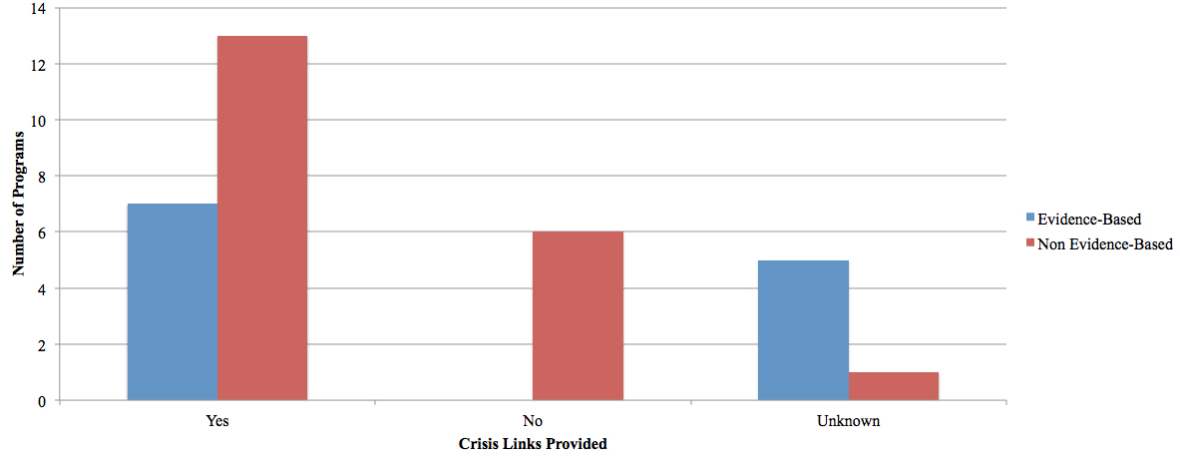
Crisis link information provided.

Crisis links were defined as email addresses, phone numbers, and/or hotlines connected to distress centers providing counseling services to at-risk users. If present, telephone numbers and distress centers were from within the program’s country of origin. In total, 20 programs (both EBP and NBP) had a crisis link with contact information and phone numbers. Most EBP provided this service (five programs were unknown); however, six NBP did not (one program unknown). See Figure 10.

### Research and Publications

Only 12 of the 32 programs had at least one published RCT evaluating their efficacy. It was beyond the scope of the current review to summarize the results of these trials and the reader is directed to recently published systematic reviews and meta-analyses [32-34] (see Multimedia Appendix 4).

## Discussion

### Principal Findings

Use and wide spread dissemination of Web-based mental health care interventions is expanding and reflected by the number of currently available depression treatment programs captured in this scoping review. Web-based approaches may have several benefits beyond those of conventional psychotherapy [36-38]. Such approaches (1) are accessible at any time of day, unlike a traditional clinic setting with predetermined hours of operation, (2) are accessible from home therefore removing transportation barriers to treatment and health care provider shortages (particularly in rural communities), (3) are cost effective and reach a mass audience, (4) are self-guided by the user, and (5) allow the user to remain anonymous while in the setting of their choice (privacy). However, it is important to note that Web-based approaches may have several disadvantages including the potential perpetuation of secrecy associated with mental illness and depriving the individual of the opportunity for face-to-face interaction afforded by traditional therapeutic techniques.

We have identified 32 existing interactive Web-based programs and have found varying degrees of accessibility, quality, and evidence supporting their efficacy. Only 12 of the 32 programs had at least one peer-reviewed, published article describing the results of an efficacy study. In examining the programs, authors noted a number of similarities and differences when comparing EBP to NBP by each evaluation point (see Multimedia Appendix 3). Using the adapted evaluation criteria [35], each program was rated using 28 categories. Totals for a few EBP and NBP were amended if a score could not be provided for a program in the category, as indicated by a superscript “a” (^a^) (ie, the information was not available on the program website and/or inquiries to program developers were not returned). EBP had a smaller range in scores (68%-92%) and a higher overall average score (80%) relative to the wider range for NBP (39%-92%) and a lower overall average score (73%). While generally comparable, there were some subtle differences between EBP and NBP programs on the evaluation criteria of accessibility, program structure, use of validated assessment tools, additional features, peer support forums, safeguards, and the provision of user-statistics as further discussed below.

While Web-based programs can generally improve access to mental health care, some aspects of existing programs may present users with alternative access barriers. For example, some programs were available only in one language (eg, Interapy and Kleur je Leven in Dutch, and Internetpsykiatri in Swedish), with the majority of NBP available only in English. The addition of alternative languages could promote open accessibility to any user seeking treatment. Furthermore, some programs had accessibility restrictions based on country of residence (see Multimedia Appendix 2), which may have been due to limited therapist availability for those programs providing clinician support. Given the global nature of the Internet, consideration could be made to avoid geographic restrictions on program availability.

While a higher proportion of the NBP (9/19^a^; 47%) were freely available (no fees or referrals required to access) as compared to the EBP (3/10^a^; 30%), a higher proportion of the NBP (6/19^a^; 32%) than the EBP (3/10^a^; 30%) had a fee for accessing the program. Requirements such as therapist referral, administrator acceptance for registration, and/or user fees may act as deterrents to use as they necessitate additional motivation and resources on the part of the user. The need to obtain a referral, enter personal information to register, and/or wait for access negates the benefits of anonymity and convenience afforded by Web-based tools. Conversely, registration requirements enable the user to track their progress and build on previously completed modules. In addition, the registration of personal information would allow the program deliverers to contact the user when in need, such as when increases in depression symptoms or suicide risk (which are more prevalent among depressed individuals as compared to other mental health disorders) are reported. Also, referral-based programs often allowed for integrated therapist contact. For fee-based programs, free demonstration/trial modules could be provided to allow users to assess the program prior to making a financial commitment.

In examining the programs captured in the review, CBT was the most commonly incorporated therapeutic approach (EBP=6, NBP=9; see Table 3). Other therapeutic techniques included psychoeducation, IPT, positive psychology, and narrative therapy. Recent evidence suggests that Web-delivered, self-guided IPT is as effective as Web-delivered CBT on symptoms of depression in general community samples [39]. Future investigations could continue to examine which techniques are most efficiently and effectively delivered through the Web and whether the mode of delivery (eg, text, audio, video) differentially affects outcome. In regards to program content and structure (evaluation point 11, ie, guided [modules must be completed in a set order] vs unguided [any module is accessible at any time]), 80% (8/10^a^) of EBP offered the guided approach relative to 63% (12/19^a^) of the NBP. Future studies should evaluate the relative effectiveness of guided versus unguided interventions.

Many of the programs captured in this review delivered treatment specifically for adolescents, adults, or both. A limited number of programs catered to special populations (eg, military personnel, older adults). Future programs could be geared toward the needs of special populations such as individuals with cognitive impairments or persons in a caregiving role. Accommodations for cognitively impaired individuals may include larger text sizing, multimodal delivery (audio and video files), in addition to programming specific to their impairments (eg, memory games, goal setting and problem solving). Caregivers of chronically ill patients also demonstrate increased psychological distress and burden [40,41]. Apart from depression, increased caregiver anxiety, guilt, rage, grief, substance abuse, and elevated risk of relapsing into a pre-existing mood disorder has been noted among caregivers [39,42,43]. Future programs could be tailored to meet the issues faced by caregivers in day-to-day life, including an emphasis on preserving and increasing available family resources in caregiving circumstances [44]. Program developers should use feedback from members of these populations to fully understand learning preferences as well as accessibility issues specific to these individuals.

Although a target audience was identified for each of the programs (ie, adults, students, special population), course content was often generic for all users within the targeted population. Personalized treatment plans (ie, generic programs vs individual treatment plans; evaluation point 12) were offered in only a few programs: 22% of EBP (2/9^a^) and NBP (4/18^a^). Personalized treatment plans may enhance user engagement by appealing to their specific treatment needs and offering relevant treatment information. Take for example a user suffering from only a mild form of depression; they may not have found additional anxiety information useful causing them to lose interest in continuing with the program despite its potential benefit. In programs offering a personalized treatment plan, program suggestions were based on an initial assessment. All but a few EBP and NBP offered assessments; however, not all of the assessment tools used were validated (evaluation point 15). Of EBP, 75% (6/8^a^) relative to 67% (12/18^a^) of NBP employed validated assessment tools (ie, BDI, PHQ-9, or CES-D). Programs should strive to offer validated assessment tools to provide users with accurate feedback in regards to their depressive and anxiety symptoms. During this emotionally sensitive time period, individuals could be heavily influenced by program feedback and results, necessitating accurate and valid depictions of depression symptoms over time.

In addition to worksheets and assessments, some programs offered additional features that may help enhance usage and retention including emails offering encouragement, helpful quotes or testimonials, and reminders to complete modules; completion trackers for each session and/or the program overall; supplementary worksheets and mood assessments delivered during or after each session to assess and monitor progress; and automated feedback to the user. The majority of both EBP (7/10^a^; 70%) and NBP (10/20; 50%) offered three or more of these additional features (see Table 4). The effect of these features on user satisfaction and treatment efficacy should be further investigated.

Many of the programs included additional integrated therapist contact, peer support discussion forums, and crisis links. Programs offering therapist support (evaluation point 21) were delivered via telephone, video conference, or live chat (ie, instant messaging): 60% (6/10^a^) of EBP and 50% (8/19^a^) of NBP offered therapist support. Similarly, 60% (6/10^a^) of EBP and 50% (8/19^a^) of NBP provided a therapist name and their credentials (evaluation point 22). Providing users with this information may provide them peace of mind that they are being cared for and monitored by an accredited individual capable of intervening if required. A recent study using MoodGYM plus brief face-to-face therapist support indicated positive results in the reduction of symptoms of depression in a primary care setting [45]. Although additional therapist support has been shown to be effective [45,46], further investigation is needed to understand therapist user interaction impact on patient outreach, treatment experience, and concept reinforcement. Conversely, efficacy has also been demonstrated in programs that did not include clinician support [47,48]. When choosing an intervention program, this option is influenced by user learning needs and should be indicated by the user. Facilitating therapist-user communication through technology could aid in maintaining user anonymity and privacy. Potential negative implications of therapist contact include reduced availability, reduced user independence, and increased pressure to complete program requirements.

Other avenues of support offered within the evaluated programs included peer discussion forums, blogs, and shared user spaces (evaluation point 23). Unlike EBP, none of which provided peer-support forums, 44% (8/18^a^) of NBP offered this feature. However, only 24% (4/17^a^) of NBP offered forums that were monitored by an overseeing authority, facilitating safe user interaction and positive constructive topics of conversation (evaluation point 24). Peer support offers a level of familiarity not offered with clinician support. The need for relatedness to others enduring similar emotional issues can be both comforting and motivating; however, the effectiveness of peer support upon symptom resolution has yet to be evaluated in this context.

In addition to peer and clinician support, some programs offered crisis links via telephone hotlines, email, or chat functions. Hotlines provided support to users under distress when therapists or other social support options were unavailable. Safeguards (evaluation point 17) were available in all the evaluated EBP; however, only 68% (13/19^a^) of NBP offered this feature. Due to the sensitive nature of the treatment and topics discussed, all programs should offer or provide information for available crisis links.

To evaluate program usability, we contacted each of the program’s administrators. Many were unable or did not wish to disclose user statistics in regards to registration, attrition, and program completion. Those that responded to inquiry emails or posted statistics on their program websites are listed in Table 3. Among the few programs providing data (EBP, 7/10^a^ or 70%; NBP, 10/19^a^ or 53%), statistics varied greatly. Examining the existing literature, completion rates in RCTs of Web-delivered treatments for depression have primarily ranged from 55%-67% [49-54] with rates as low as 20% [55]. Future research should examine which aspects of a program could promote retention and completion such as email reminders or motivators/incentives. End-user feedback may be useful in identifying less effective areas within programs and facilitate modification.

Although not included in this review (as they did not meet the inclusion and exclusion criteria), three novel and noteworthy Web-based treatment programs for depression were identified: Depression Quest [56], Moodbuster [57], and SPARX [58]. Depression Quest and SPARX are gaming programs that deliver innovative depression treatments to adolescents. Depression Quest invites the user to experience life from the perspective of a depressed individual. Users read scenarios, select one of the decisions provided, and navigate the path associated with their choice. Program developers aimed to (1) aid caregivers by providing insight into the depressed mindset, and (2) demonstrate to individuals affected by depression that they are not alone in their struggle. In SPARX, users navigate an avatar of their choice towards an end objective while fighting gloomy negative automatic thoughts along the way. Targeting depression, anxiety, and stress, clinical trials of the program have demonstrated positive results on symptoms of depression [59]. The third program, Moodbuster, provides an interactive treatment program similar to those included in the review; however, it also incorporates the use of biosensors worn on the body throughout the day. Biosensors are equipped to transmit information on emotionally influenced bodily responses like electrodermal activity, respiration, and electrocardiography changes. A monitoring system for medication intake is also provided to users prescribed pharmacological treatment. Sensors are set to monitor dose and intake information. With all sensors feeding back to the program, Moodbuster interprets the information and reasons which type of therapy is most likely to be effective. Although resource intensive, a program like Moodbuster may be effective for depression resistant to alternative forms of treatment; however, research is needed to further evaluate this treatment approach.

**Figure 11:**
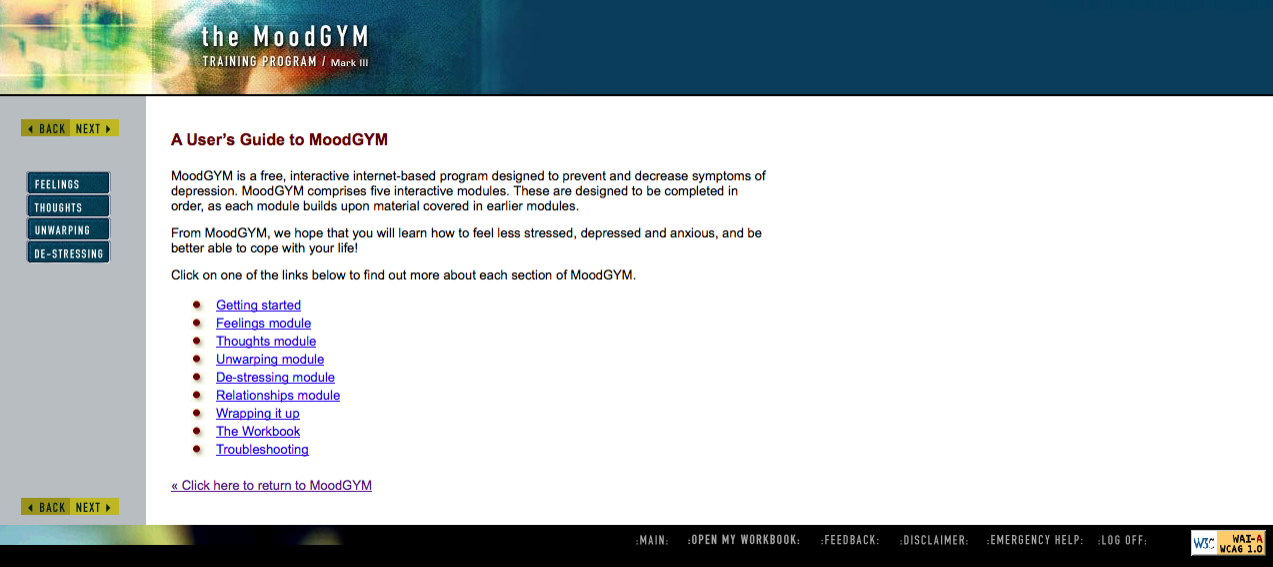
Mood Gym introduction page.

**Figure 12:**
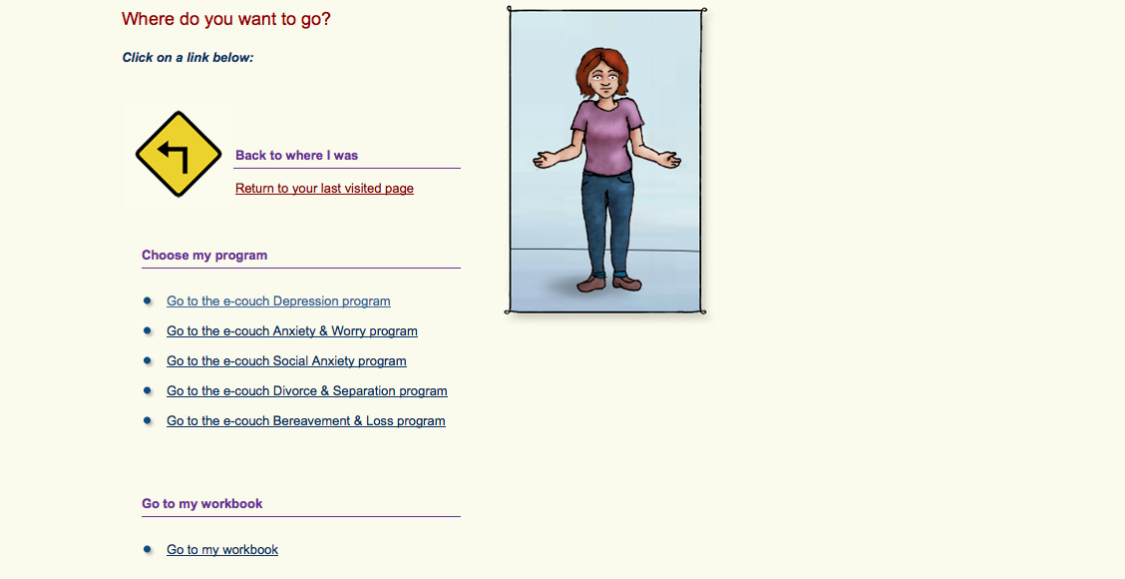
E Couch navigation page.

**Figure 13:**
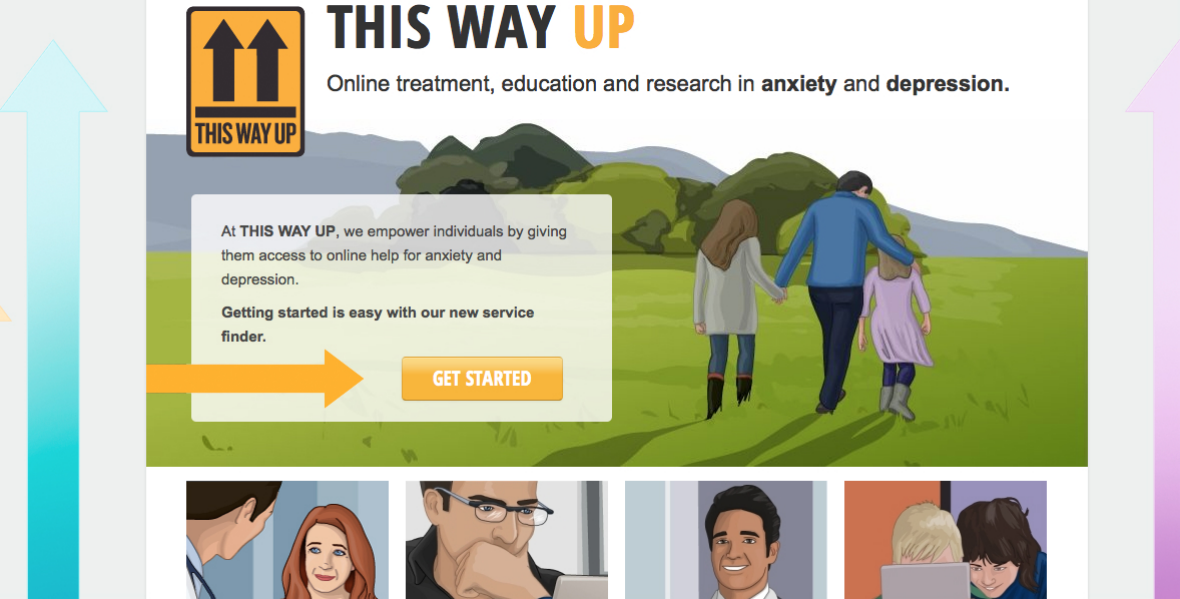
This Way Up welcome page.

In summary, many interactive treatment programs for depression are available on the Web; however, the efficacy and validity of most of these programs (20/32, 63%) have not been evaluated using RCTs. When comparing those programs that are evidence-based to those that have not been empirically evaluated, more of the EBP programs seemed to use a guided approach, employ validated assessment tools, offer additional features, incorporate safeguards, and provide user statistics. More of the NBP programs were available without fees or referrals (however, a higher proportion did request a user fee than the EBP) and offered peer-support forums. Based on our review, several programs emerged that are easily accessible, free to use, and have supporting evidence for their efficacy including E Couch, MoodGYM, and This Way Up (Self Help Course, Worry and Sadness; see Figure 11-13 for screenshots of these programs).

Although there is a strong and growing body of evidence in support of Web-based interventions, some perceive that the uptake and dissemination of such programs have not been commensurate with their potential to improve health-related outcomes. With respect to Web-based interventions for depression, potential barriers have been cited such as negative clinician and patient attitudes [60,61], legal and ethical regulations related to online clinician-patient interactions [62], a lack of practitioner willingness to refer patients to such interventions, and clinician fears of losing work [63]. In order to increase implementation and reach across a range of settings, Burnett and Glasgow have suggested using tailored messaging and social networking functionality, in particular leveraging newer technologies that offer novel ways for users to store, view, manipulate, share, and experience their personal data (eg, Web 2.0 design principles) [64].

### Limitations

While we aimed to be comprehensive, systematic, and thorough in our review and evaluation, some information could not be ascertained due to referral and cost restrictions limiting program accessibility. We used the Canadian version of common search engines (Google, Yahoo, and Bing) to identify programs conforming to evaluation inclusion criteria. Searches were repeated until results became redundant; however, it is possible that despite our efforts to be inclusive, some programs were missed. It is also important to note that searches conducted in other countries (using the above mentioned search engines) may not yield the same search results. Provided that many search engines suggest local websites and may also receive funding from local advertisers, our search results may be highly specific to our proximity (Toronto, Canada). Moreover, the rapidly evolving nature of the Internet means that the same search conducted several months from now could yield a different set of results and conclusions. New programs may be developed, existing programs may be discontinued, adapted, or amalgamated, and new research trials could support or refute the efficacy of these programs.

It should also be noted that programs were evaluated from the perspective of a researcher and not of an end-user. A person with depression, for example, may have different opinions regarding the usability and quality of the identified programs. The intended end-user would likely provide developers with a more subjective opinion. Programs may also be biased to the type of user they capture, that is, individuals with less severe forms of depression. Individuals suffering from more severe forms of depression may face greater decreases in motivation and are less likely to access and participate in an intervention program. Consequently, users with severe forms of depression may be underrepresented in RCTs and in collected user feedback and data. Feedback is necessary to facilitate change as well as outline likes and dislikes for various features. Overall, end-user input should be sought as it is crucial to improving treatment delivery and program functionality.

### Future Directions

At this time, there are few programs available for special populations (eg, caregivers, individuals with cognitive deficits, older adults). It is important that program functionality accommodate accessibility of varying populations to ensure adequate treatment is delivered. Studies investigating the needs of these special populations could inform the development of new programs and the adaptation of existing ones. Future programs could also aim for increased accessibility. This could entail multilanguage delivery, elimination of residency restrictions, elimination of registration fees or referrals, flexibility in module timing, and minimization of mandatory user response (eg, mandatory worksheets and mood assessments).

With respect to research, although the minority of the identified programs were evidence-based as defined by the presence of at least one evaluative RCT, the trials that have been completed to date have been generally of strong quality with adequate sample sizes [32-34]. In addition to testing the efficacy of these programs, future research should examine the barriers, facilitators, and modifiers to program uptake, compliance, and retention. The impact of aspects such as program structure and content, registration requirements, user fees, therapist support, and specific features (eg, reminders, feedback, peer-support groups) on program efficacy, compliance, retention, safety, and cost-effectiveness require further investigation. In addition, factors related to treatment response sustainability need to be examined.

### Conclusions

In this review, we identified 27 websites offering 32 programs with interactive components aimed at reducing symptoms of depression among users. The programs varied widely in terms of content, accessibility, usability, method of delivery (eg, text, audio, or video), and supplementary tools. A minority of the programs identified had empiric evidence to support their efficacy for the treatment of depressive symptoms.

In choosing to use or refer a Web-based treatment program for depression, the user may wish to consider the following factors: ease of use and accessibility, availability of additional features and support needs, and most critically, programs that have been validated with good quality research. Users are encouraged to critically evaluate their program choice and should investigate research supporting program claims.

Web-delivered interventions afford many potential advantages to individuals. Users can log on to their preferred program in the comfort of their own home 24 hours a day, 365 days a year. This may help to increase accessibility, reduce prolonged wait times, and address privacy concerns. Furthermore, it is potentially cost-efficient and convenient, allowing users to seek treatment when desired. Developers should continue to create such programs and tailor additional sites to the needs of specialty groups.

## Multimedia Appendix 1

Search log.

## Multimedia Appendix 2

Accessibility of the programs evaluated.

## Multimedia Appendix 3

Program evaluation log.

## Multimedia Appendix 4

Randomized controlled trial citations for evidence-based programs.

## Multimedia Appendix 5

Program screenshots.

## Abbreviations

CBT: cognitive behavioral therapy
EBP: evidence-based program
IPT: interpersonal therapy
NBP: non-evidence-based program
RCT: randomized controlled trial
