# Supplementary material for: Web-Based Intervention Programs for Depression: A Scoping Review and Evaluation
Source: J Med Internet Res. 2014 Sep 23;16(9):e209. doi: 10.2196/jmir.3147 (PMC4211022; doi:10.2196/jmir.3147)
Supplement: Supplementary file 4 [file jmir_v16i9e209_app4.docx]

| Ref # | Randomized Controlled Trial Citation |
| --- | --- |
| 1 | 1.Proudfoot J, Goldberg D, Mann A, Everitt B, Marks I, Gray J. Computerized, interactive, multimedia cognitive behavioural therapy reduces anxiety and depression in general practice: a randomised controlled trial. Psychological Medicine 2003a;33:217-227. PMID:12622301.; 2.Proudfoot J, Ryden C, Everitt B, Shapiro D, Goldberg D, Mann A, Tylee A, Marks I, Gray JA. Clinical efficacy of computerised cognitive-behavioural therapy for anxiety and depression in primary care: randomised controlled trial. British Journal of Psychiatry 2004;185:46-54. PMID:15231555.; 3.Grime P. Computerised cognitive-behavioural therapy at work: A randomised controlled trial in employees with recent stress-related absenteeism. Occupational Medicine 2004;54:353-359. PMID:15289593. |
| 2 | 1.Billings DW, Cook RF, Hendrickson A, Dove DC. A web-based approach to managing stress and mood disorders in the workforce. J Occup Environ Med 2008;50:960-968. PMID:18695455. |
| 3 | 1.Meyer B, Berger T, Caspar F, Beevers CG, Andersson G, Weiss M. Effectiveness of a novel integrative online treatment for depression (Deprexis) : Randomized controlled trial. J Med Internet Res 2009;11(2):e15. PMID:19632969.; 2.Berger T, Hammeril K, Gubser N, Andersson G, Caspar F. Randomized controlled trial comparing guided with unguided self-help. Cognitive Behaviour Therapy 2011;40(1):251-266. PMID:22060248.; 3.Moritz S, Schilling L, Hauschildt M, Schroder J, Treszi A. A randomized controlled trial of internet-based therapy in depression. Behaviour Research and Therapy 2012;50(7-8):513-521. PMID:22677231. |
| 4 | 1.Titov N, Dear BF, Schwencke G, Andrews A, Johnston L, Craske MG, McEvoy P. Transdiagnositic internet treatment for anxiety and depression : a randomized controlled trial. Behaviour Research and Therapy 2011;49(8):441-452. PMID:21679925.; 2.Titov N, Dear BF, Johnston L, McEvoy PM, Wootton B, Terides MD, Gandy M, Fogliati V, Kayrouz R, Rapee RM. Improving adherence and clinical outcomes in self-guided internet treatment for anxiety and depression: a 12-month follow-up of a randomised controlled trial. PLoS One 2014;9(2):e89591. PMID: 24586897. |
| 5 | 1.Donkin L, Hickie IB, Christensen H, Naismith SL, Neal B, Cockayne NL, Glozier N. Rethinking the dose-response relationship between usage and outcome in an online intervention for depression : randomized controlled trial. J Med Internet Res 2013; 15(10):e231. PMID:24135213.; 2.Donker T, Batterham PJ, Warmerdam L, Bennett K, Bennett A, Cuijpers P, Griffiths KM, Christensen H. Predictors and moderators of response to internet-delivered Interpersonal Psychotherapy and Cognitive Behaviour Therapy for depression. J Affect Disord 2013;151(1):343-51. PMID:23953024.; 3. Glozier N, Christensen H, Naismith S, Cockayne N, Donkin L, Neal B, Mackinnon A, Hickie I. Internet-delivered cognitive behaviour therapy for adults with mild to moderate depression and high cardiovascular disease risks: a randomised attention-controlled trial. PLoS One 2013;8(3):e59139. PMID:23555624. |
| 6 | 1.Wright JH, Wright AS, Albano AM, Basco MR, Goldsmith LJ, Raffield T, Otto MW. Computer-assisted cognitive therapy for depression : maintaining efficacy while reducing therapist time. Am J Psychiatry 2005;162(6):1158-1164. PMID:15930065. |
| 7 | 1.Ruwaard J, Schrieken B, Schrijver M, Broeksteeg J, Dekker J, Vermeulen H, and Lange A. Standardized web-based cognitive behavioural therapy of mild to moderate depression: a randomized controlled trial with a long-term follow-up. Cognitive Behaviour Therapy 2009;38(4):206-221. PMID:19221919. |
| 8 | 1.de Graaf LE, Gerhards SA, Arntz A, Riper H, Metsemakers JF, Evers SM, Severens JL, Widdershoven G, Huibers MJ. Clinical effectiveness of online computerized cognitive behavioural therapy without support for depression in primary care: a randomized trial. British Journal of Psychiatry 2009;195(1):73-80. PMID:19567900.; 2.de Graaf LE, Hollon SD, Huibers MJ. Predicting outcome in computerized cognitive behavioral therapy for depression in primary care: A randomized trial. Journal of Consulting and Clinical Psychology 2010;78(2):184-189. PMID:20350029.; 3.Spek V, Nyklicek I, Smits N, Cuijpers P, Riper H, Keyzer J, Pop V. Internet-based cognitive behavioural therapy for subthreshold depression in people over 50 years old: A randomized controlled clinical trial. Psychological Medicine 2007; 37(12):1797-1806. PMID:17466110.; 4.Spek V, Cuijpers P, Nyklicek I, Smits N, Riper H, Keyzer J, Pop V. One-year follow-up results of a randomized controlled clinical trial on internet-based cognitive behavioural therapy for subthreshold depression in people over 50 years. Psychological Medicine 2008;38(5):635-639. PMID:18205965.; 5.Warmerdam L, van Straten A, Twisk J, Riper H, Cuijpers P. Internet-based treatment for adults with depressive symptoms: randomized controlled trial. Journal of Medical Internet Research 2008;10(4):e44. PMID:19033149.; 6.Warmerdam L, van Straten A, Jongsma J, Twisk J, Cuijpers P. Online cognitive behavioral therapy and problem-solving for depression symptoms: Exploring mechanisms of change. J Behav Ther Exp Psychiatry 2010;41(1):64-70. PMID:19913781. |
| 9 | 1.Calear AL, Christensen H, Mackinnon A, Griffiths KM. Adherence to the MoodGYM program: outcomes and predictors for an adolescent school-based population. J Affect Disord 2013;147(1-3):338-44. PMID:23245469.; 2.Calear AL, Christensen H, Mackinnon A, Griffiths KM, O’Kearney R. The YouthMood Project: a cluster randomized controlled trial of an online cognitive behaviour program with adolescents. J Consult Clin Psychol 2009;77(6): 1021-32. PMID:19968379.; 3.O’Kearney R, Kang K, Christensen H, Griffiths K. A controlled trial of a school-based internet program for reducing depressive symptoms in adolescent girls. Depress Anxiety 2009;26(1):65-72. PMID:18828141.; 4.O’Kearney R, Gibson M, Christensen H, Griffiths KM. Effects of a cognitive-behavioural internet program on depression, vulnerability to depression and stigma in adolescent males: a school-based controlled trial. Cogn Behav Ther 2006;35(1):43-54. PMID:16500776.; 5.Christensen H, Griffiths KM, Mackinnon AJ, & Brittliffe K. Online randomized controlled trial of brief and full cognitive behaviour therapy for depression. Psychological Medicine 2006;36(12):1737-1746. PMID:16938144.; 6.Ellis LA, Campbell AJ, Sethi S, O'Dea BM. Comparative randomized trial of an online cognitive-behavioural therapy program and an online support group for depression and anxiety. Journal of Cyber Therapy & Rehabilitation 2011;4(4):461-467. PMID: no ID.; 7.Farrer L, Christensen H, Griffiths KM, Mackinnon A. Internet-based CBT for depression with and without telephone tracking in a national helpline: randomised controlled trial. PLoS One 2011;6(11):e28099. PMID:22140514.; 8.Farrer L, Christensen H, Griffiths KM, Mackinnon A. Web-based cognitive behaviour therapy for depression with and without tracking in a national helpline: secondary outcomes from a randomized controlled trial. J Med Internet Res 2012;14(3):e68. PMID:22738715.; 9.Lintvedt OK, Griffiths KM, Sorensen K, Ostvik AR, Wang CE, Eisemann M, Waterloo K. Evaluating the effectiveness and efficacy of unguided internet-based self-help intervention for the prevention of depression: a randomized controlled trial. Clin Psychol Psychother 2013;20(1):10-27. PMID:21887811.; 10.Mackinnon A, Griffiths KM, Christensen H. Comparative randomised trial of online cognitive-behavioural therapy and information website for depression: 12 month-outcomes. Br J Psychiatry 2008;192(2):130-4. PMID:18245031. |
| 10 | 1.Clarke G, Kelleher C, Hornbrook M, DeBar L, Dickerson J, Gullion C. Randomized effectiveness trial of an internet, pure self-help, cognitive behavioral intervention for depressive symptoms in young adults. Cognitive Behaviour Therapy 2009;38(4):222-34. PMID:19440896.; 2.Clarke G, Reid E, Eubanks D, O’Connor E, DeBar LL, Kelleher C, Lynch F, Nunley S. Overcoming depression on the Internet (ODIN): a randomized controlled trial of an Internet depression skills intervention program. Journal of Medical Internet Research 2002; 4(3):e14. PMID:12554545.; 3.Clarke G, Eubanks D, Reid E, Kelleher C, O’Connor E, DeBar LL, Lynch F, Nunley S, Gullion C. Overcoming Depression on the Internet (ODIN)(2): A randomized trial of a self-help depression skills program with reminders. Journal of Medical Internet Research 2005;7(2):e16. PMID:15998607. |
| 11 | 1.Titov N, Andrews G, Davies M, McIntyre K, Robinson E, Solley K. Internet treatment for depression: a randomized controlled trial comparing clinician vs. technician assistance. PLoS ONE 2010;5(6):e10939. PMID: 20544030; 2.Perini S, Titov N, Andrews G. Clinician-assisted internet-based treatment is effective for depression: a randomized controlled trial. Australian and New Zealand Journal of Psychiatry 2009;43(6):571-578. PMID:19440890.; 3.Watts S, Mackenzie A, Thomas C, Griskaitis A, Mewton L, Williams A, Andrews G. CBT for depression : a pilot RCT comparing mobile phone vs computer. BMC Psychiatry 2013;13:49. PMID:23391304.; 4.Williams AD, Blackwell S, Mackenzie A, Holmes E, Andrews, G. Combining imagination and reason in the treatment of depression: a randomized controlled trial of internet-based cognitive bias modification and internet-CBT for depression. Journal of Consulting and Clinical Psychology 2013;81(5):793-799. PMID:23750459.; 5.Williams AD, Blackwell S, Mackenzie A, Holmes E, Andrews G. Positive imagery cognitive bias modification (CBM) and internet-based cognitive behavioural therapy (iCBT) versus control CBM and iCBT for depression: study protocol for a parallel-group randomised controlled trial. BMJ Open 2013;3(10):e004049. PMID:24171941. |
| 12 | 1.Newby JM, Mackenzie A, Williams AD, McIntyre K, Watts S, Wong N, Andrews G. Internet cognitive behavioural therapy for mixed anxiety and depression : a randomized controlled trial and evidence of effectiveness in primary care. Psychol Med 2013;43(12):2635-48. PMID:23419552. |
