# Supplementary material for: Web-Based Intervention Programs for Depression: A Scoping Review and Evaluation
Source: J Med Internet Res. 2014 Sep 23;16(9):e209. doi: 10.2196/jmir.3147 (PMC4211022; doi:10.2196/jmir.3147)
Supplement: Supplementary file 5 [file jmir_v16i9e209_app5.pdf]

<u>Program Screenshots</u>

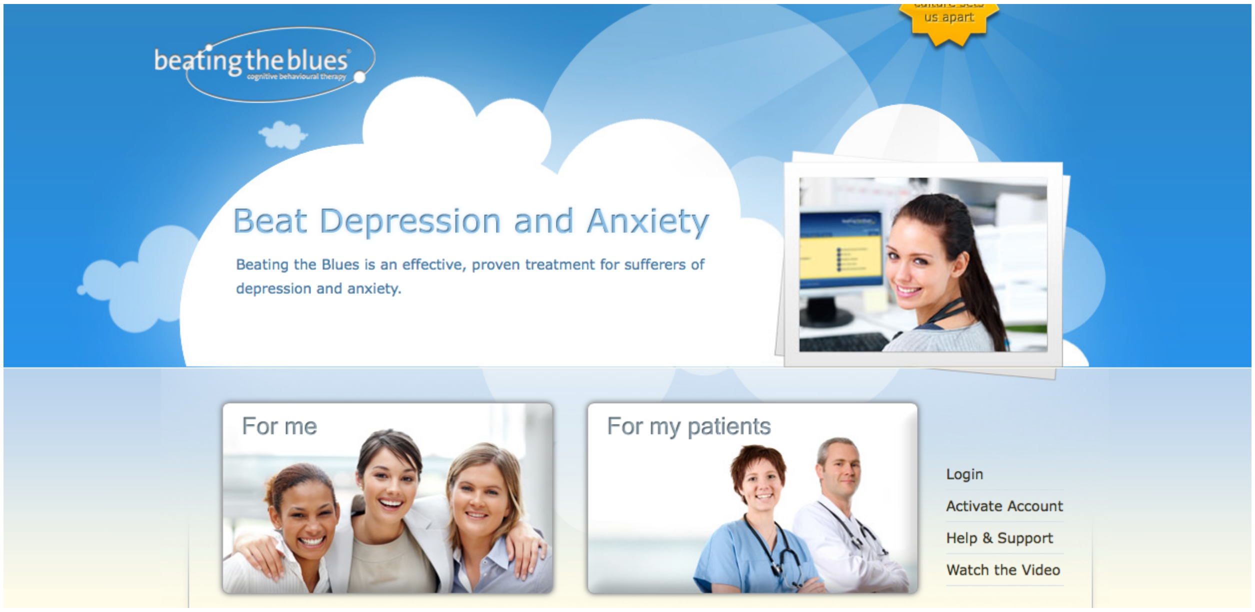

Beating the Blues welcome page.

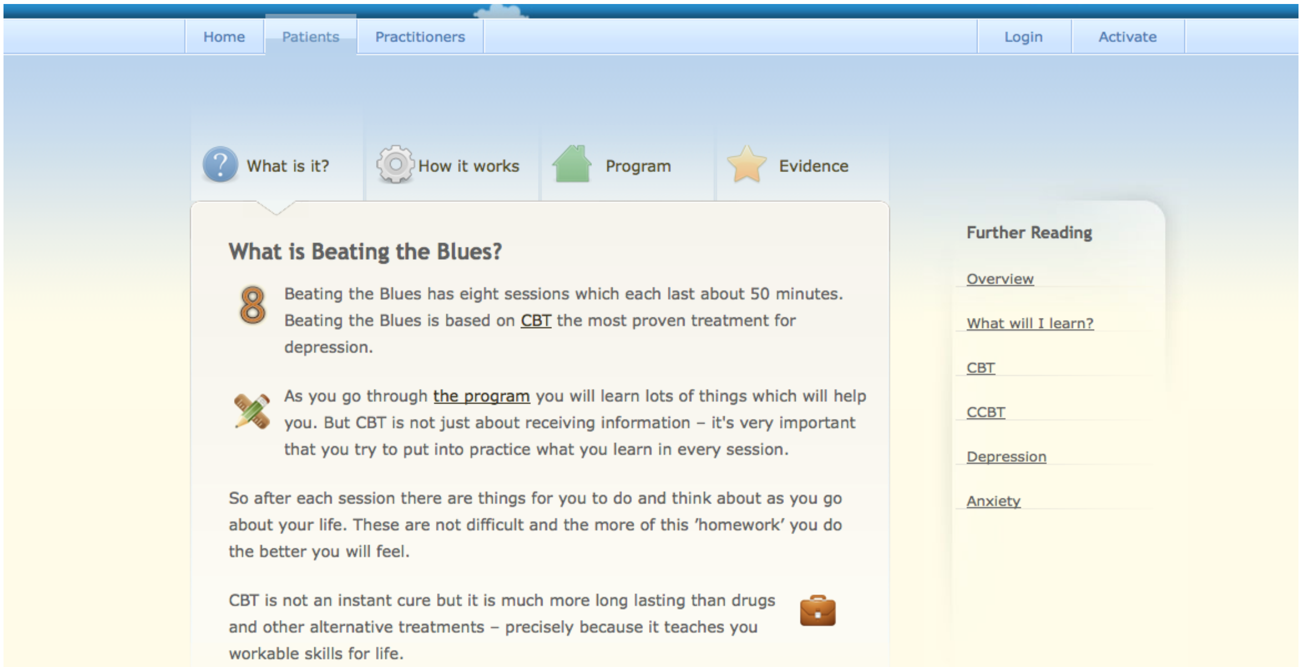

Brief program explanation for Beating the Blues users.

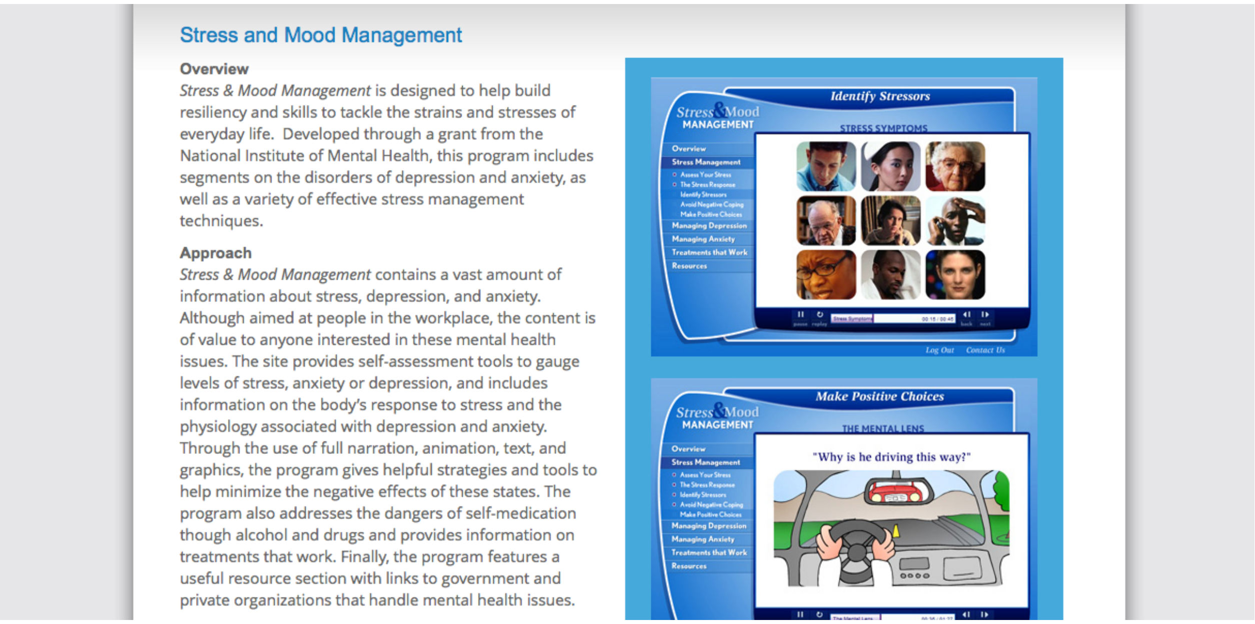

Centre for Workforce Health - Stress and Mood Management program overview.

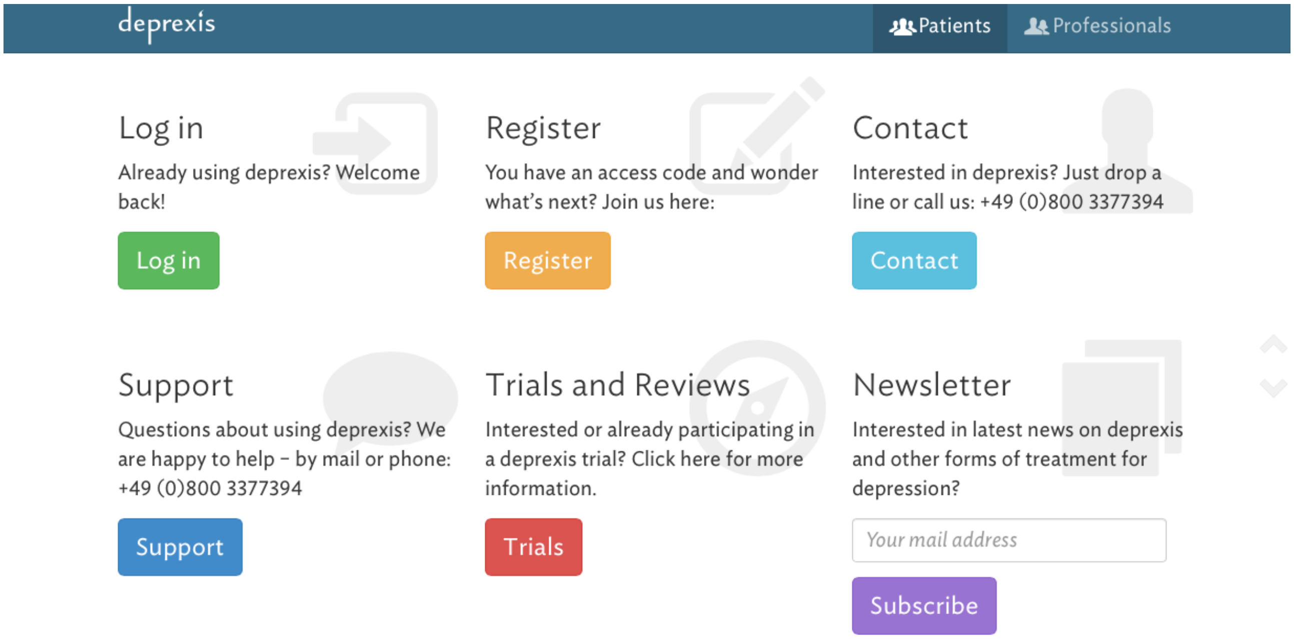

Deprexis home page navigation.

Wellbeing Course

» Wellbeing Course

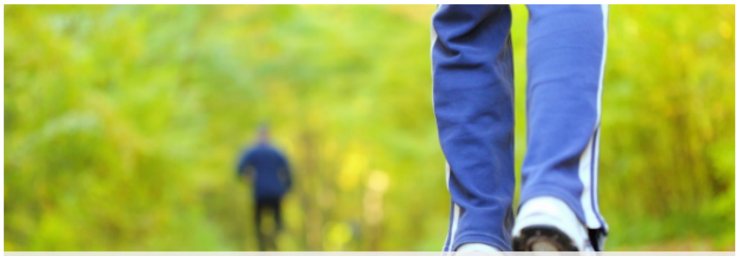

... The Wellbeing Course is designed to provide good information and teach practical, proven, skills for managing stress and anxiety, general anxiety and worry, social anxiety, panic and panic attacks and low mood and depression

LOGIN HERE!

CONTACT US ...

TAKE A TOUR ...

MEASURE SYMPTOMS ...

DONATE ...

Please like us on Facebook!

facebook

eCentreClinic - Wellbeing Course introduction.

The Wellbeing Course is our free online and internet-based treatment course designed to teach people about anxiety and depression as well as how to manage their symptoms. Anxiety and depression are common and can have a very significant impact. We developed the Wellbeing Course because many people with depression and anxiety never seek treatment. But, many have access to the internet.

Because depression and anxiety commonly occur at the same time, we thought it was important to provide information about managing each condition in the same treatment Course. So, the wellbeing is what we call a transdiagnostic treatment Course; it is designed to treat multiple conditions and types of symptoms at the same time - stress and anxiety, general anxiety and worry, social anxiety, panic and panic attacks, low mood and depression.

To learn more about symptoms of low mood and depression, please click HERE. To learn more about anxiety and anxiety disorders, please click HERE.

***PLEASE NOTE:* You can scroll right to the bottom of this page to make an application or register your interest in the Wellbeing Course.**

MACQUARIE UNIVERSITY

What does the Course involve?

- 5 online lessons that provide information and teach practical skills for managing symptoms.
- Do It Yourself (DIY) Guides to help you practice those skills.
- Lots of Additional Resources and Case Stories from others.
- Brief weekly contact with a clinician via email and telephone (we also offer the course in a self-guided format without clinician contact).

The Wellbeing Course is delivered over 8 weeks and is designed to provide you with practical information and practical, proven, skills for managing symptoms of different types of anxiety, worry, low mood and depression. The Course aims to provide the information and skills that you would normally receive from a Clinical Psychologist. We hope it will help people to gain better control over their symptoms, improve their confidence and get back to living a full and satisfying life. Importantly, the Wellbeing Course is designed

Back to Top

Wellbeing Course introduction continued.

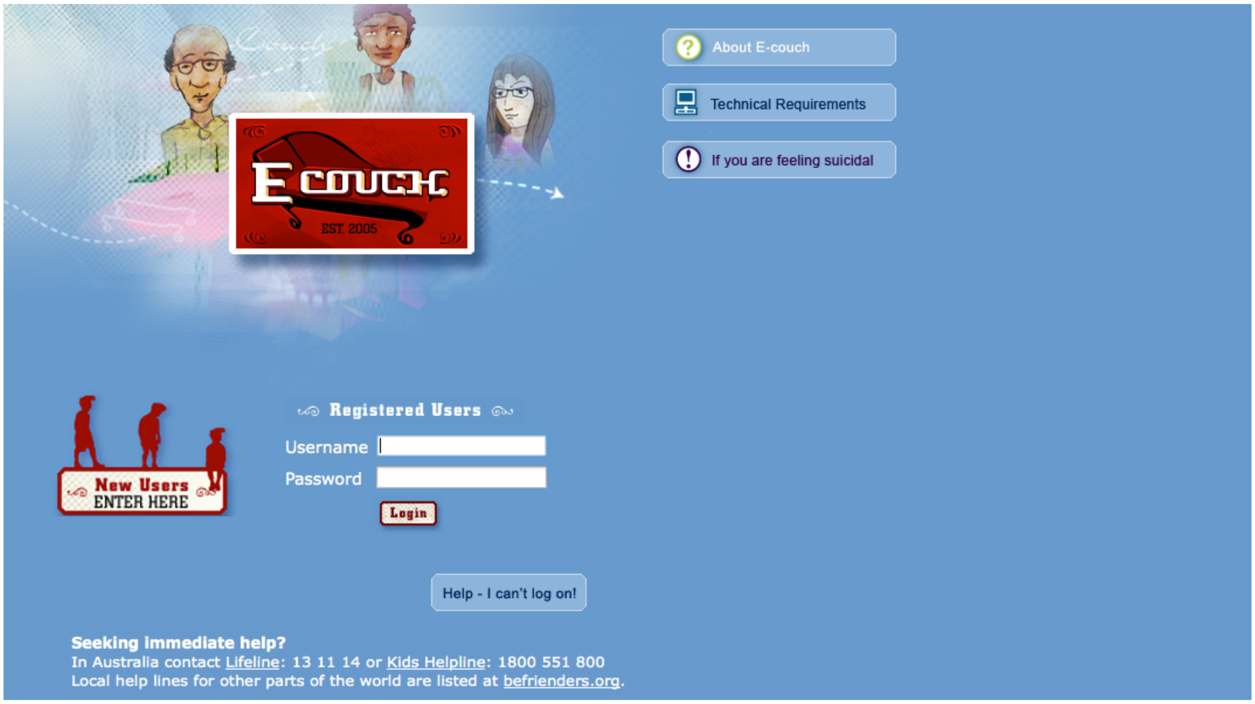

E Couch login page.

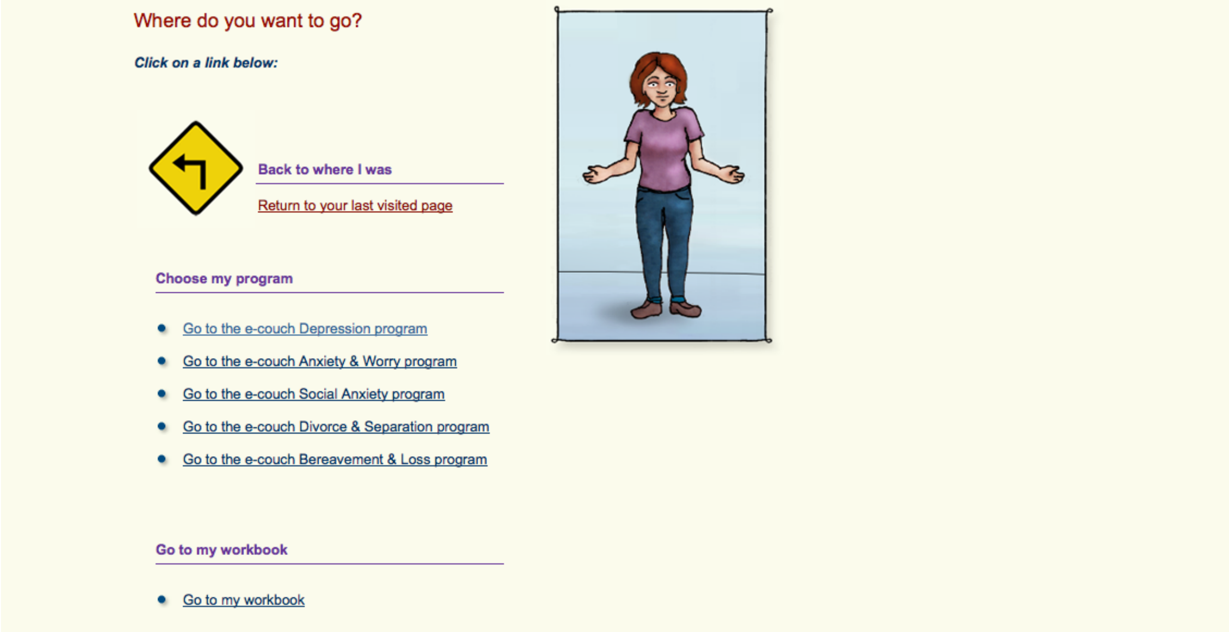

E Couch navigation page.

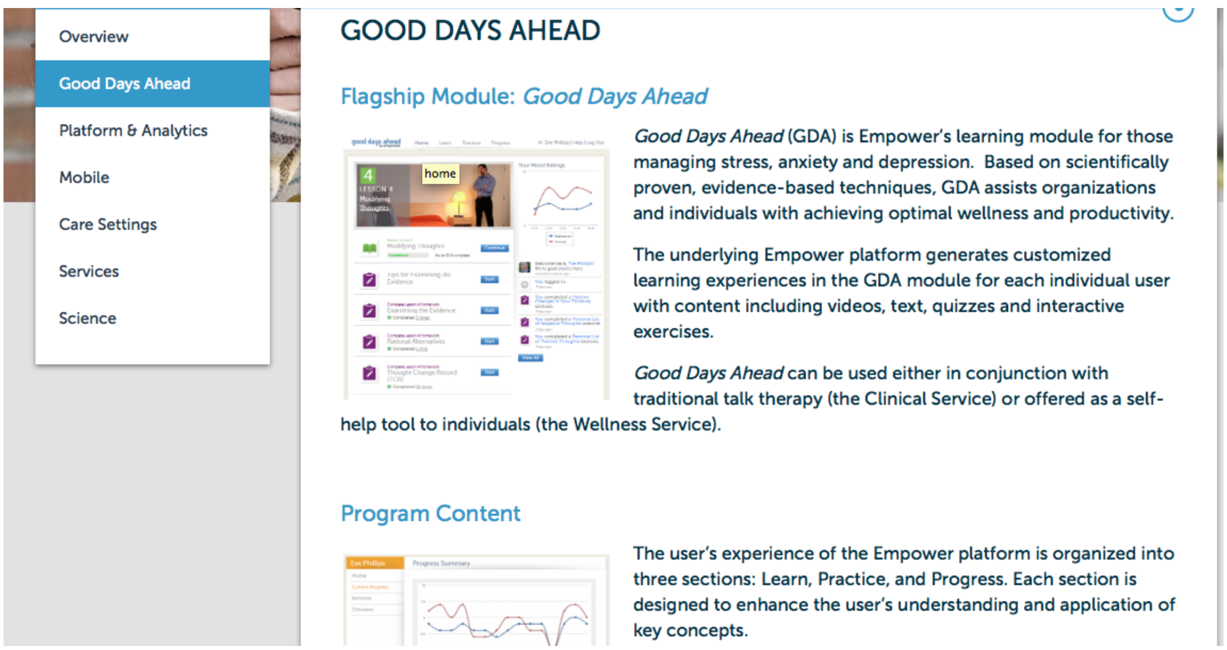

Good Days Ahead program overview.

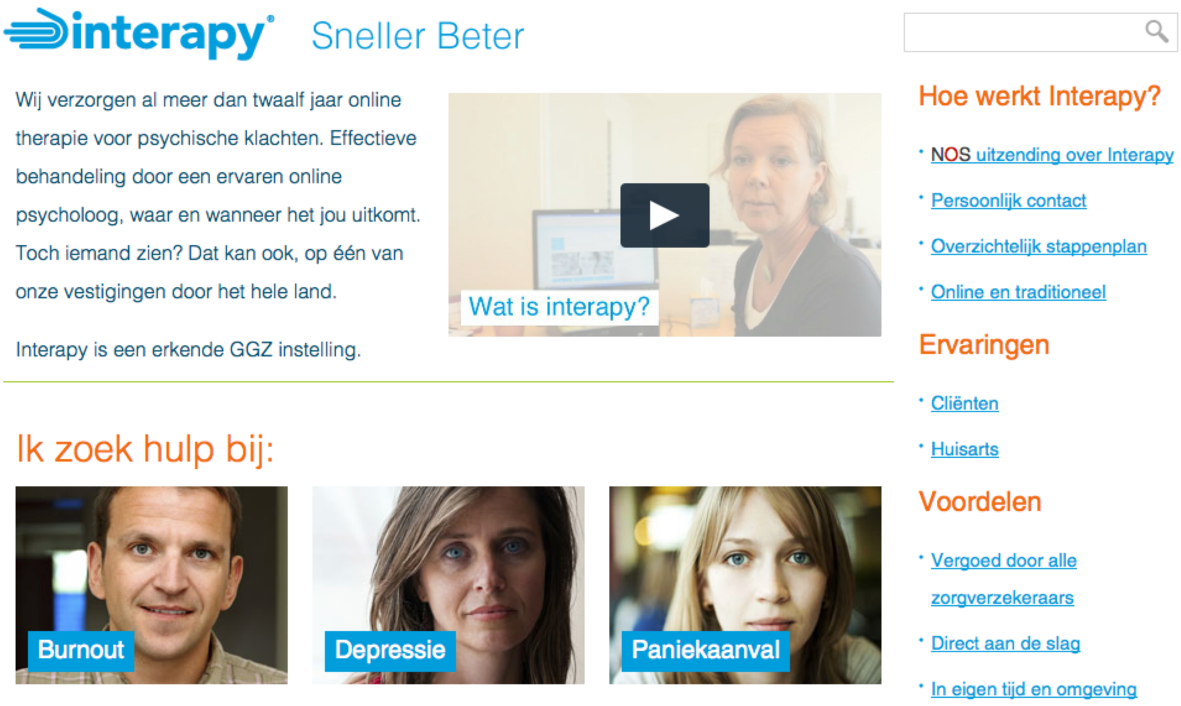

Interapy welcome page.

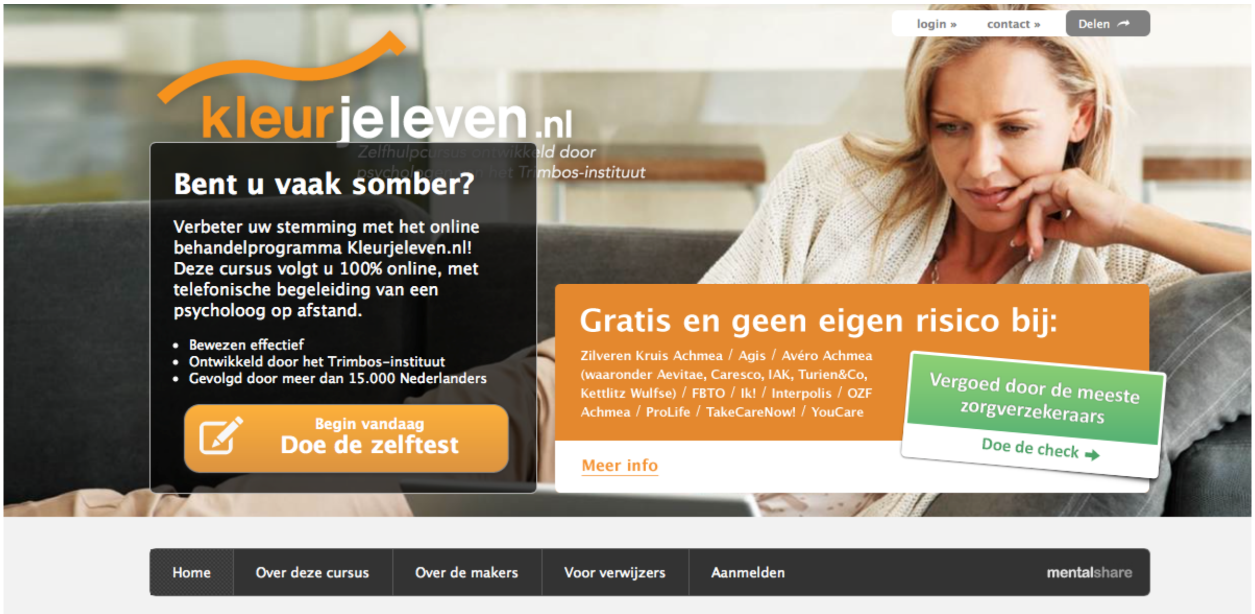

Kleur je Leven welcome page.

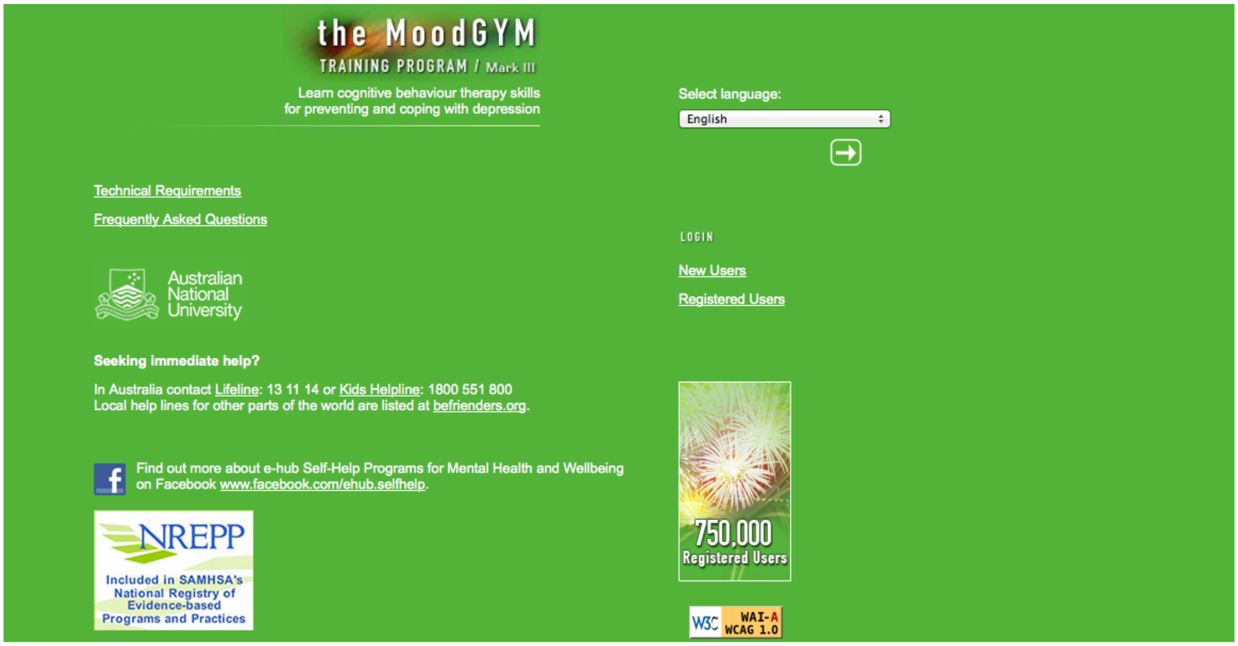

Mood Gym login page.

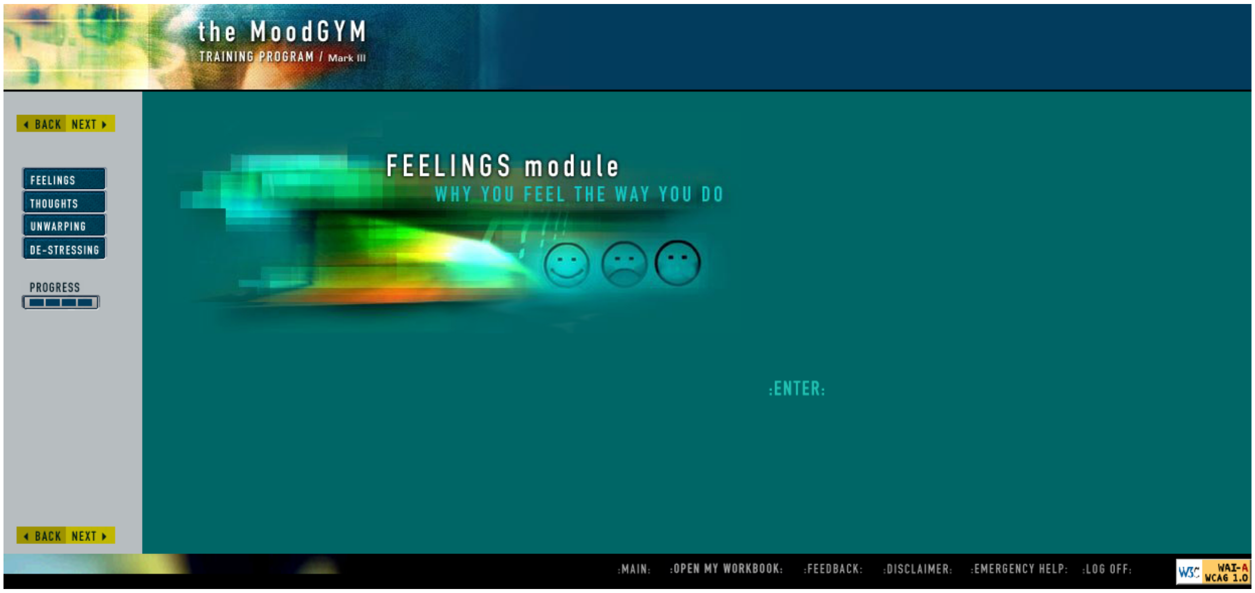

Mood Gym module welcome page.

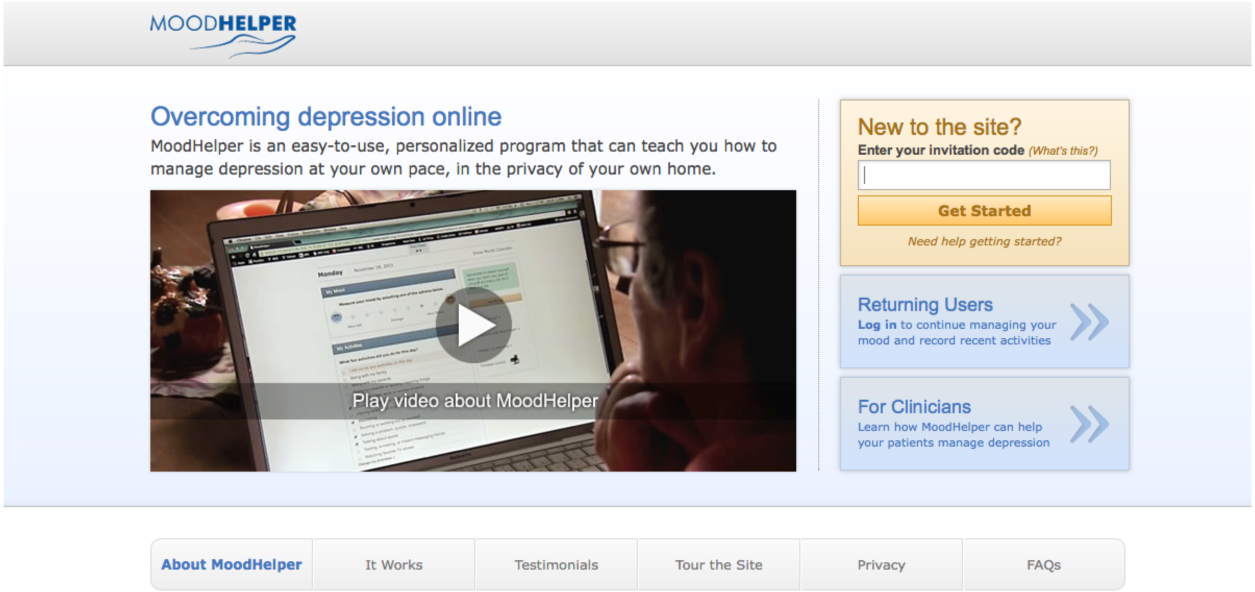

MoodHelper welcome page.

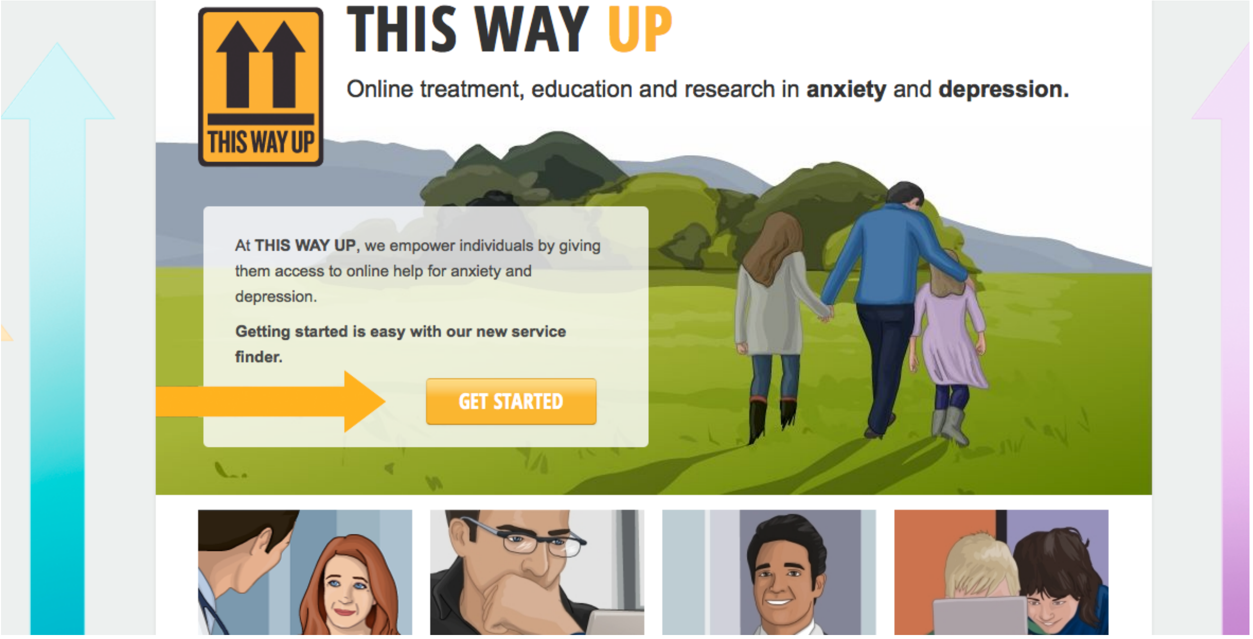

This Way Up welcome page.

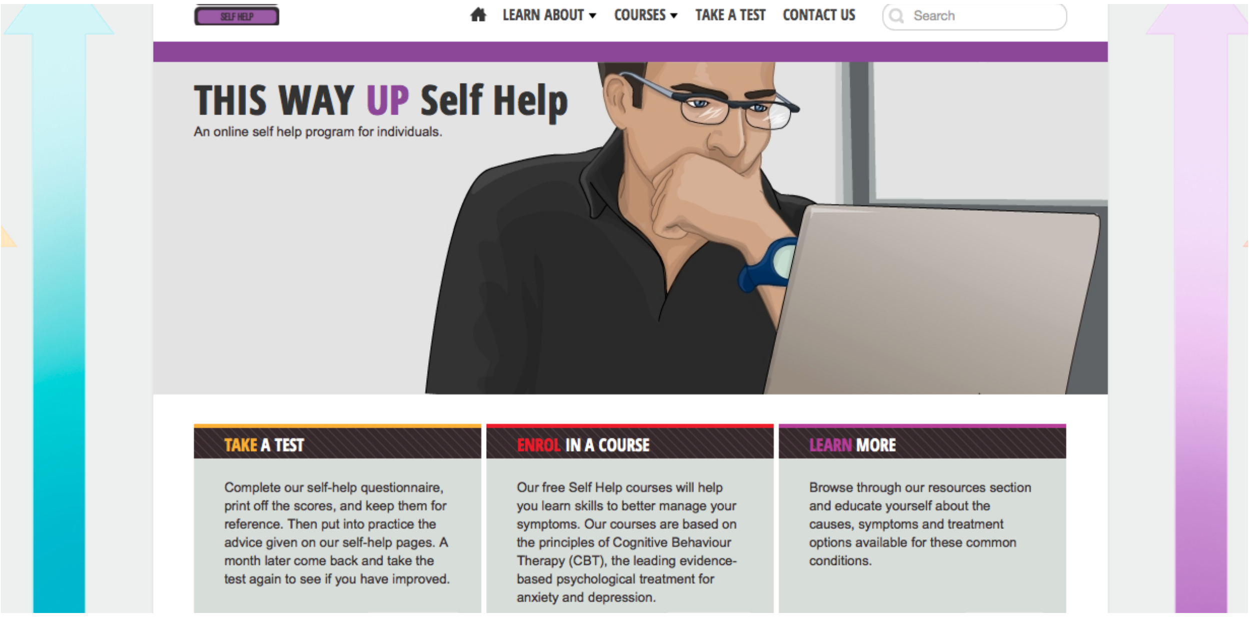

This Way Up (Self Help Course) navigation page.

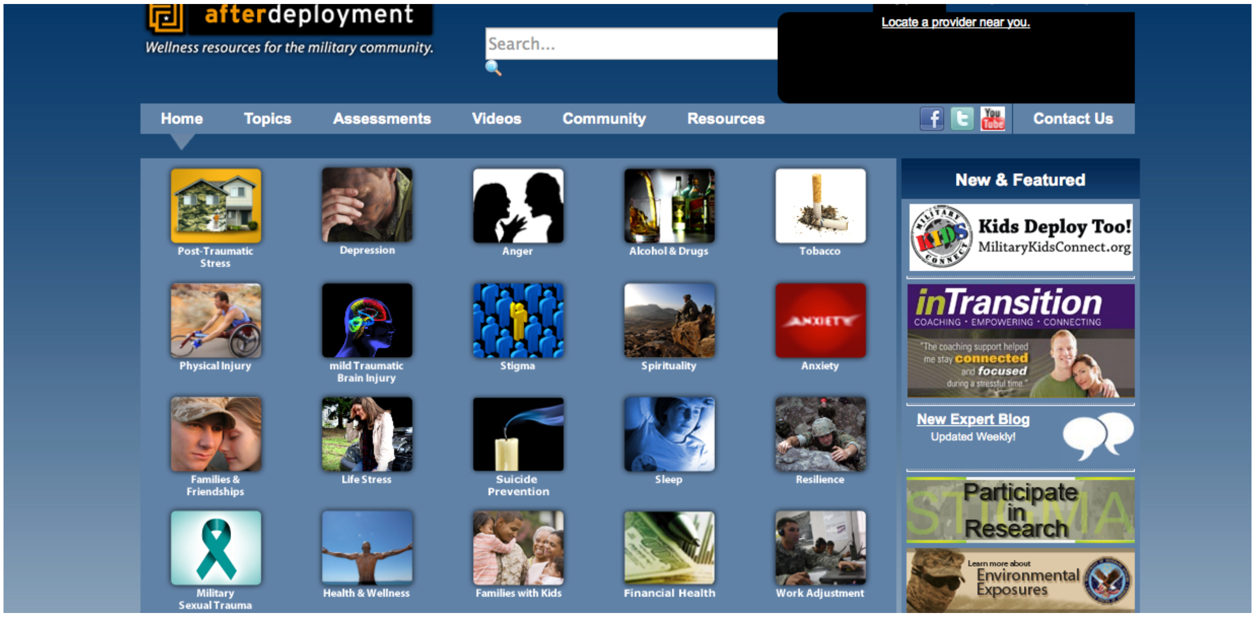

After Deployment home page navigation.

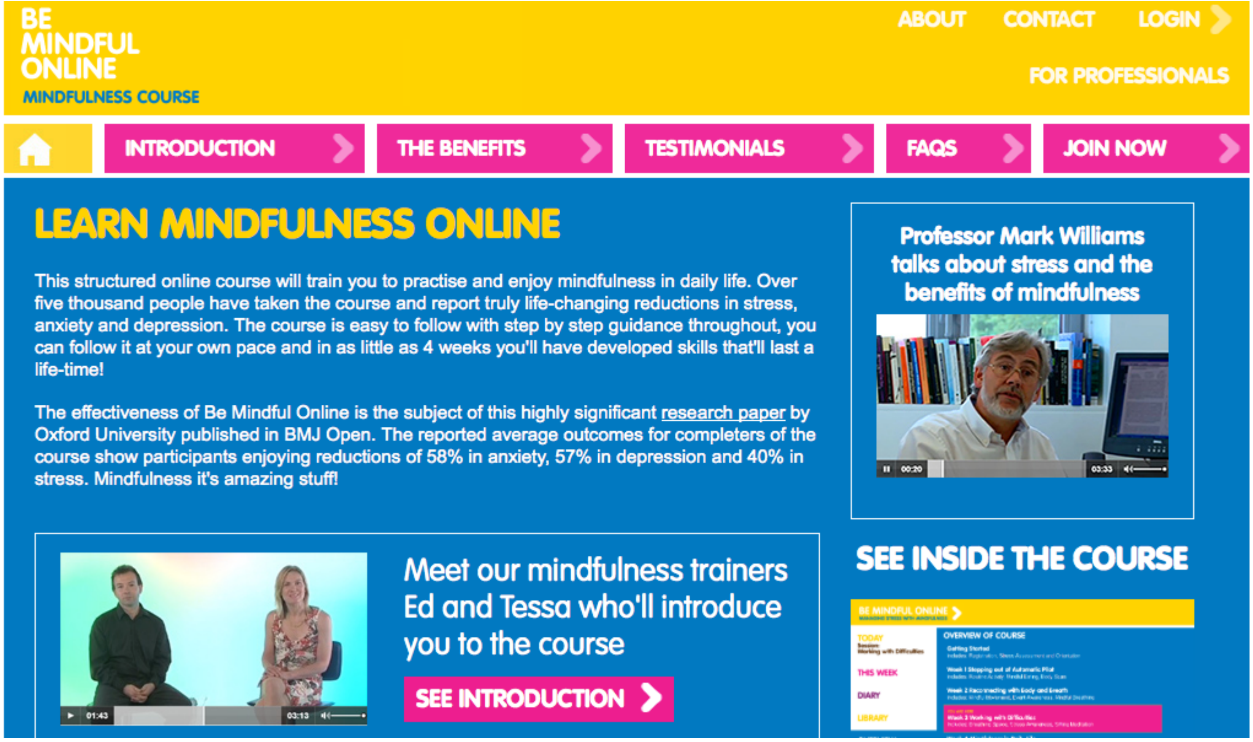

Be Mindful Online home page.

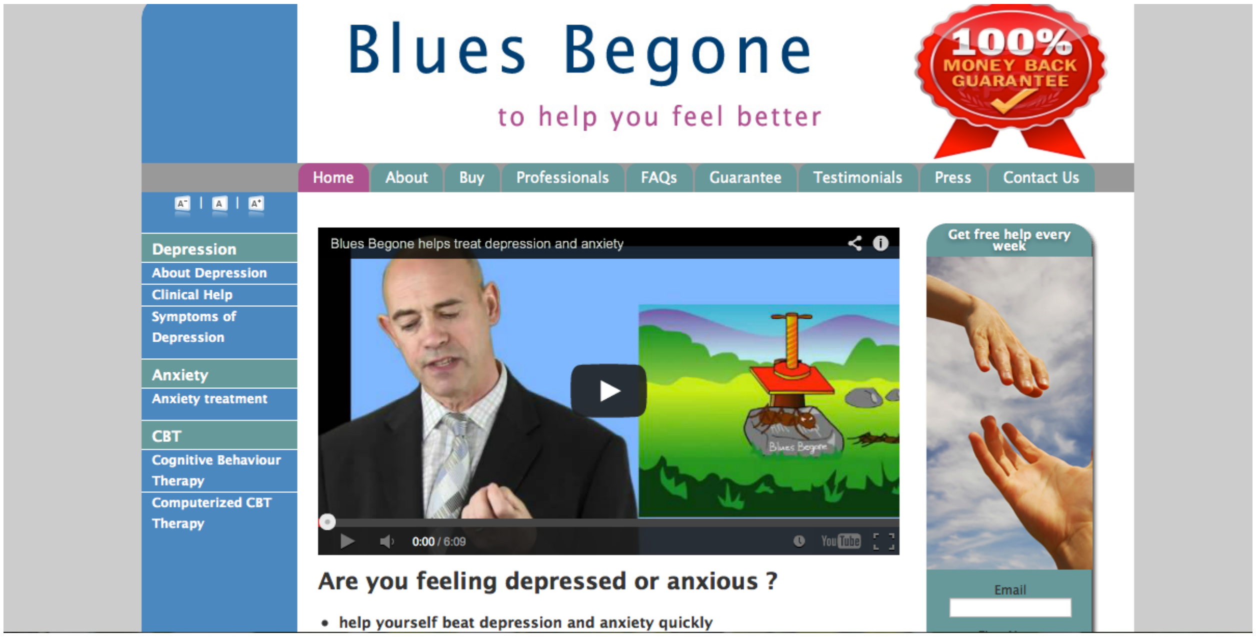

Blue Begone welcome page.

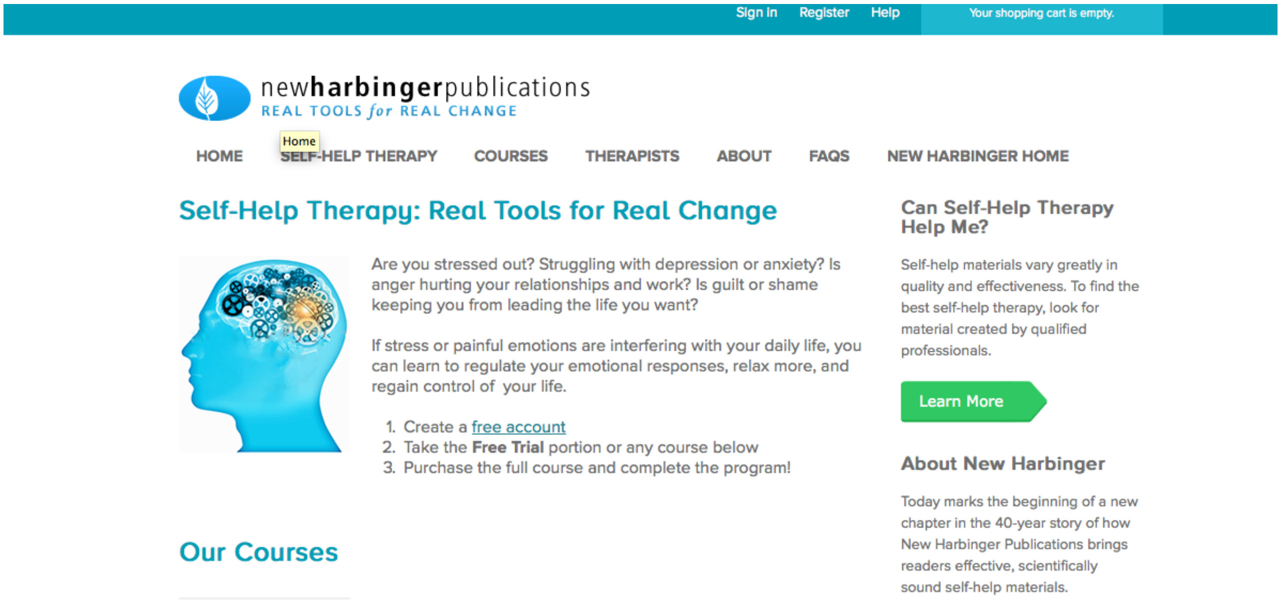

Self-Help Therapy (Mind and Emotions Course) welcome page.

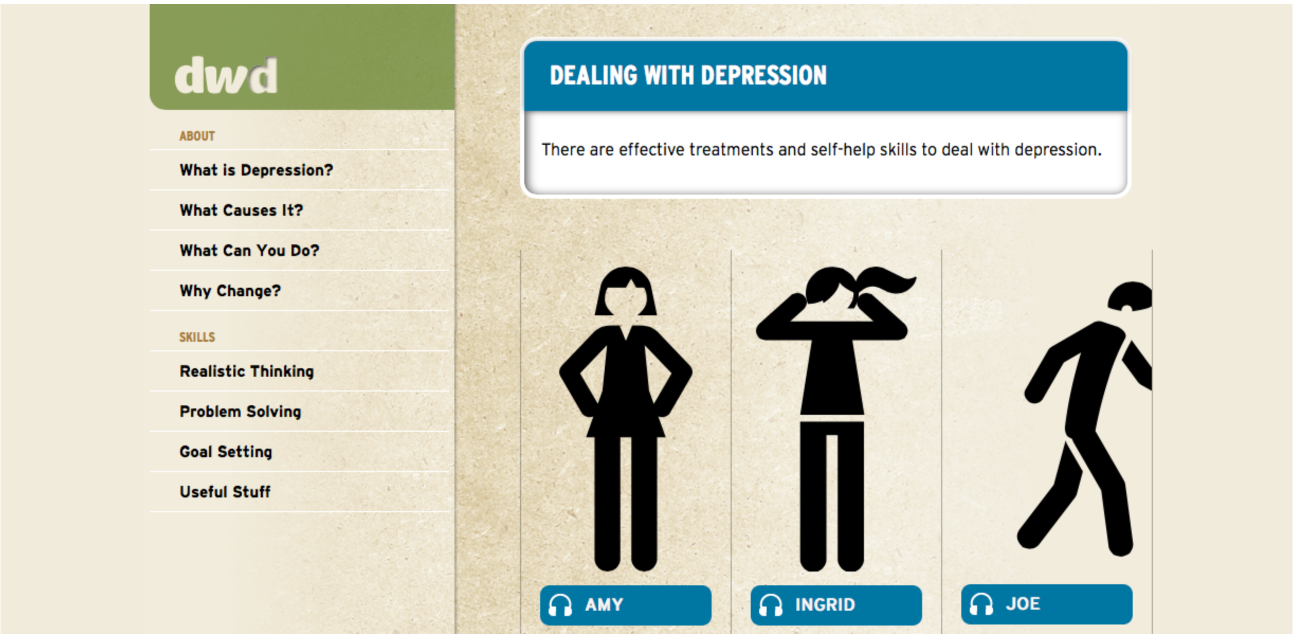

Dealing with Depression welcome page navigation.

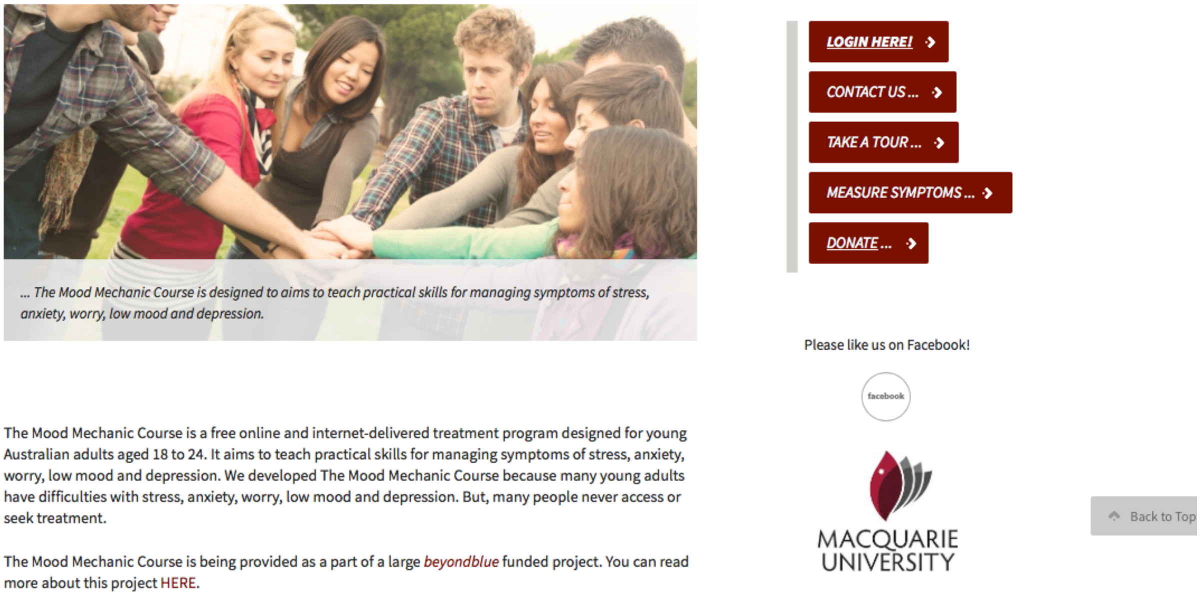

eCentreClinic - Mood Mechanic Course overview.

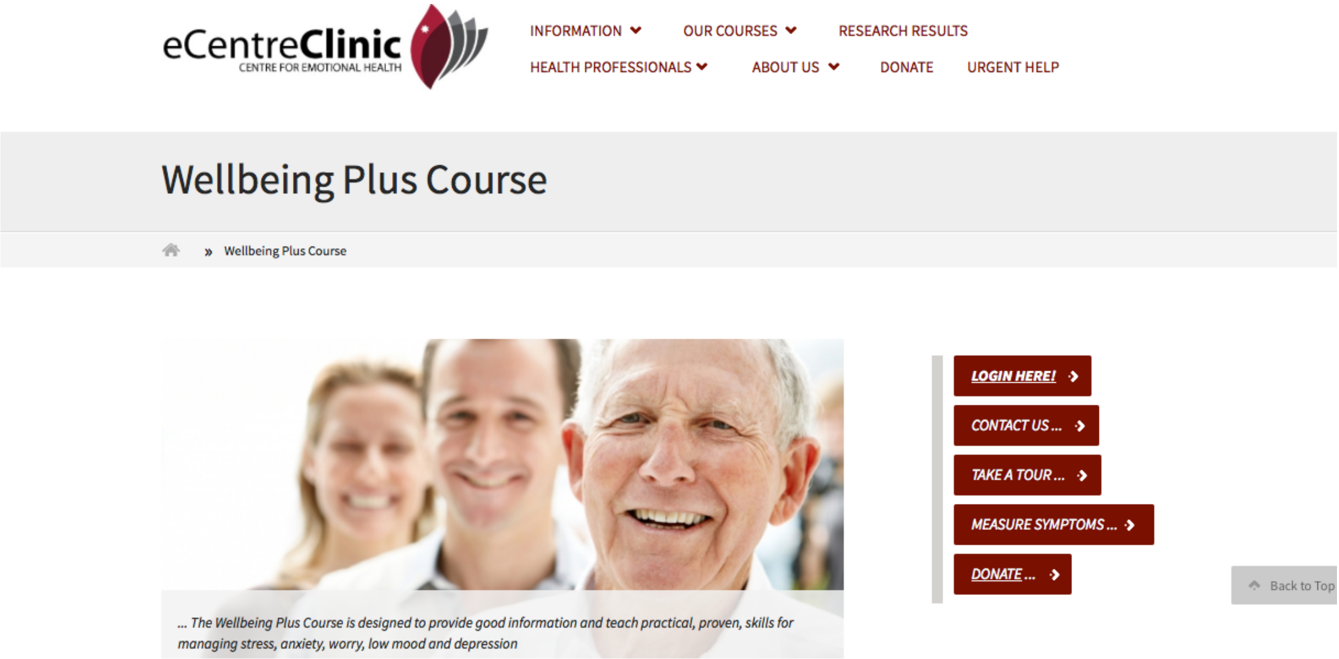

eCentreClinic - Wellbeing Plus Course introduction page.

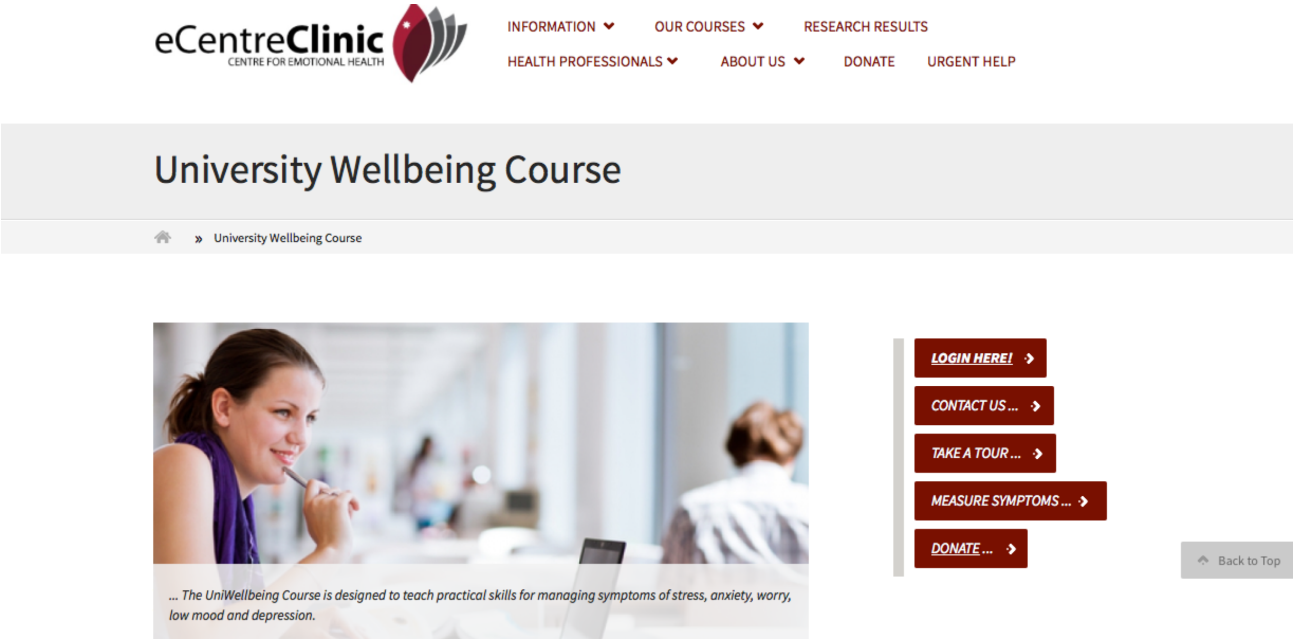

eCentreClinic - University Wellbeing Course introduction page.

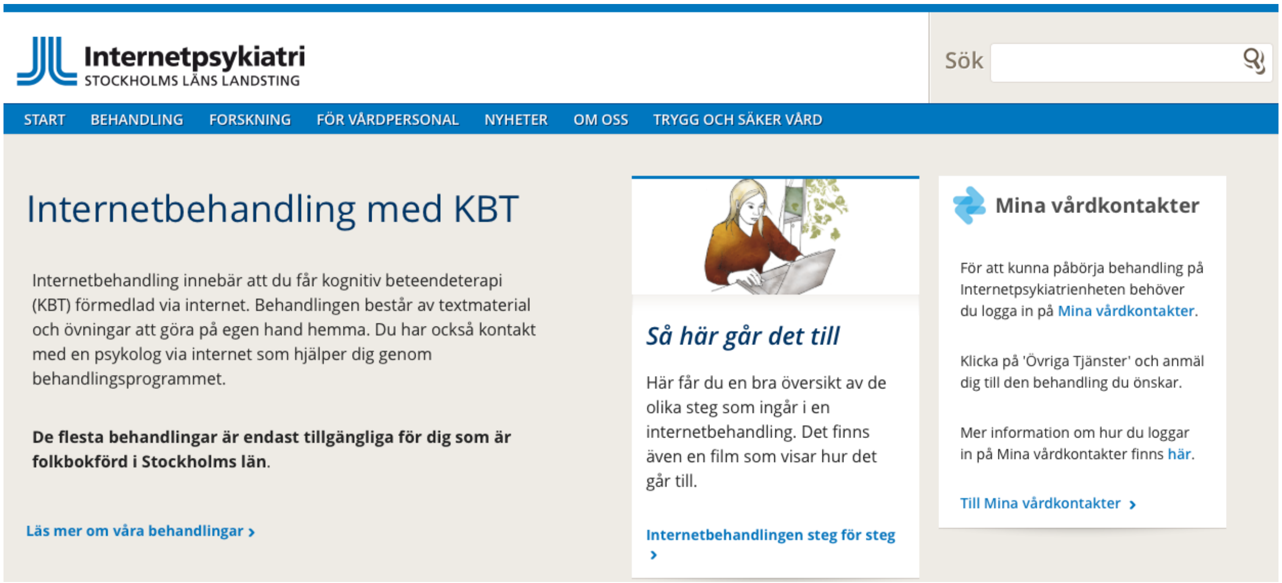

Internetpsykiatri welcome page.

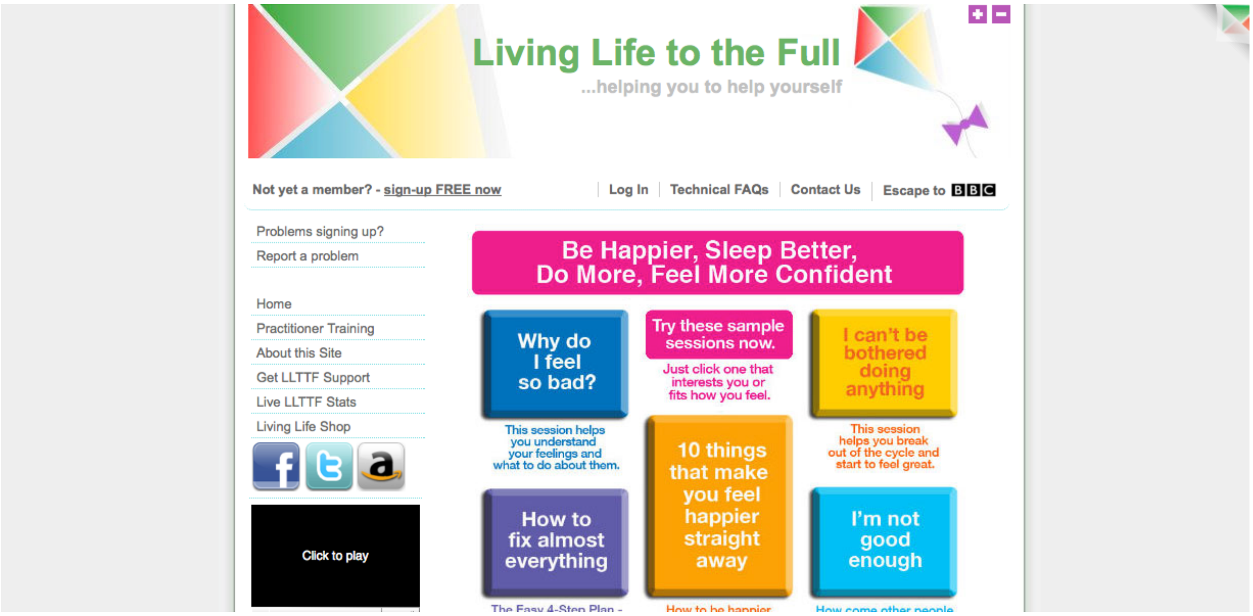

Living Life To The Full welcome page.

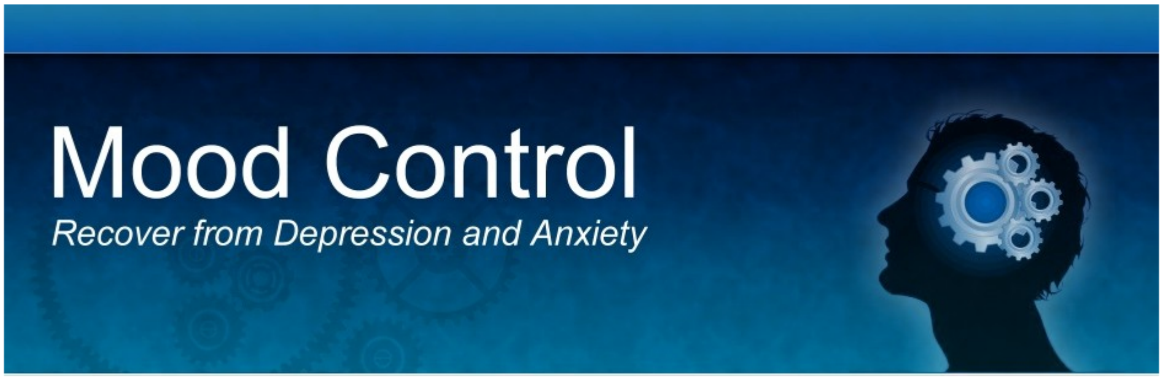

Let me explain how you can recover from depression and anxiety

Every day sufferers from depression and anxiety are able to reclaim their lives. By following a well designed program of change over a series of weeks. The neural networks that create and maintain depression and anxiety are replaced with normal and natural patterns of feelings, thoughts and behaviors. Retraining your brain like this is a reliable, repeatable and sure way to feeling better, functioning better and regaining your place in the world.

Mood Control welcome page.

Your 13 week program includes the following Mood Control coaching sessions

- Session One – Getting Started
- Session Two – External Causes
- Session Three – Life Drivers
- Session Four – Low Mood
- Session Five – Bringing it Together
- Session Six – Tolerating Uncertainty
- Session Seven – Control
- Session Eight – Increasing Confidence
- Session Nine – Faulty Thinking
- Session Ten – Attention Bias
- Session Eleven – Negative Thinking
- Session Twelve – Self Esteem
- Session Thirteen – Relapse Prevention

*And that's not all.*You'll also gain immediate access to over **200 audio-visual resources** and a wealth of **downloadable material.** You'll be in the very best hands with my **revolutionary Mood Control CBT system.** I've already seen what this system has done for **numerous patients.** I want to see you have the same results, too.

"I have worked through all of Dr Purves' excellent Mood Control sessions. I found the material extremely helpful. Time and again the practical explanations and suggestions would strike a real chord with me raising my mood and giving me insight and tools to use on an ongoing basis."

Imagine being able to try out a product without any risk. Now it's a reality!

You can access the Mood Control CBT System for just **$58 a month.** If after subscribing you are not delighted with how Mood

Mood Control module overview.

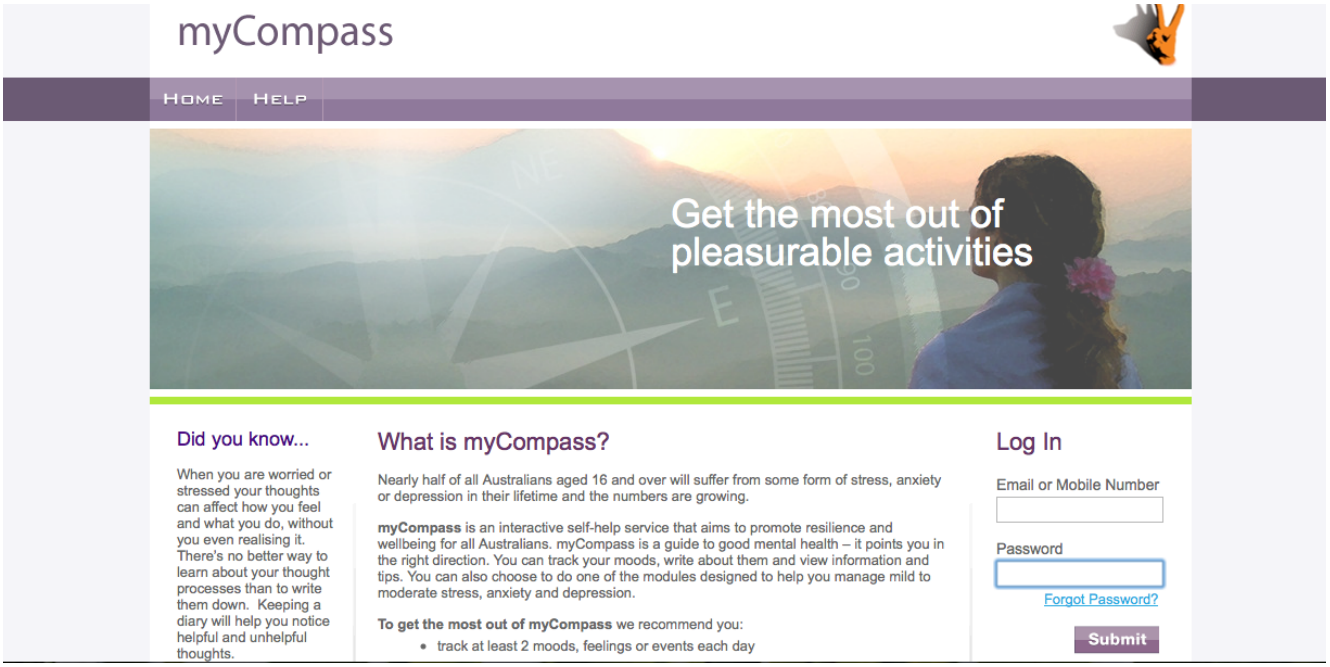

myCompass welcome and login page.

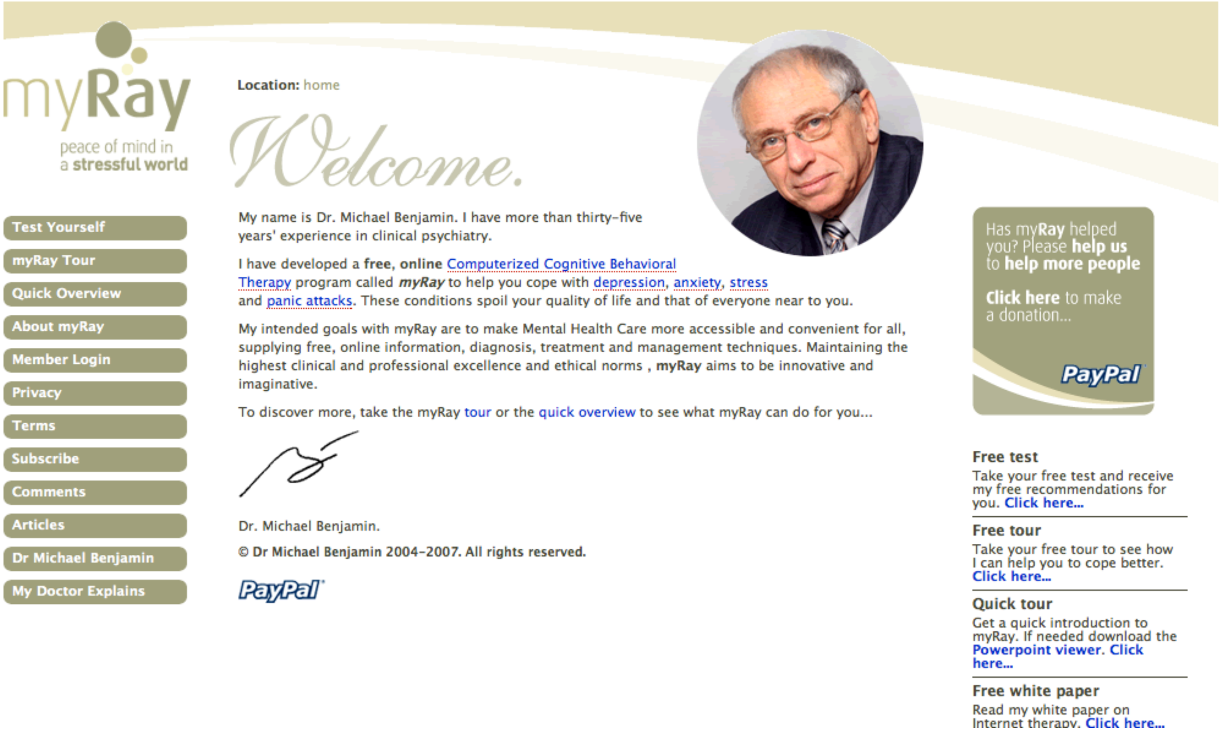

myRay welcome page.

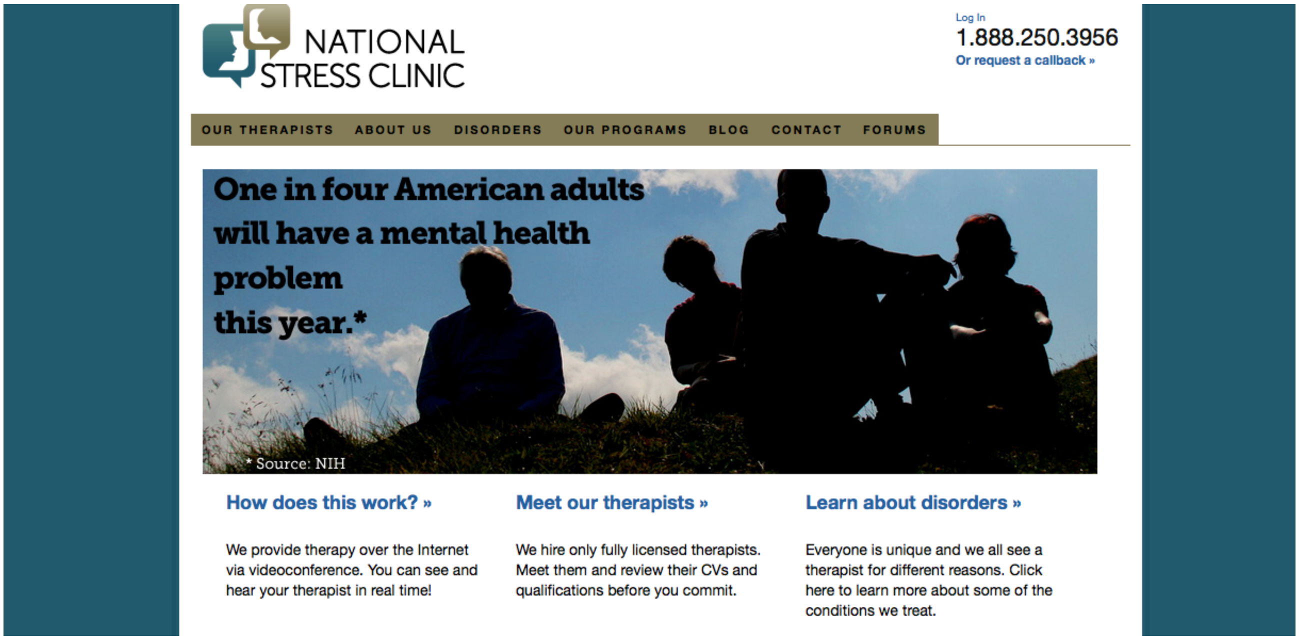

National Stress Clinic welcome page.

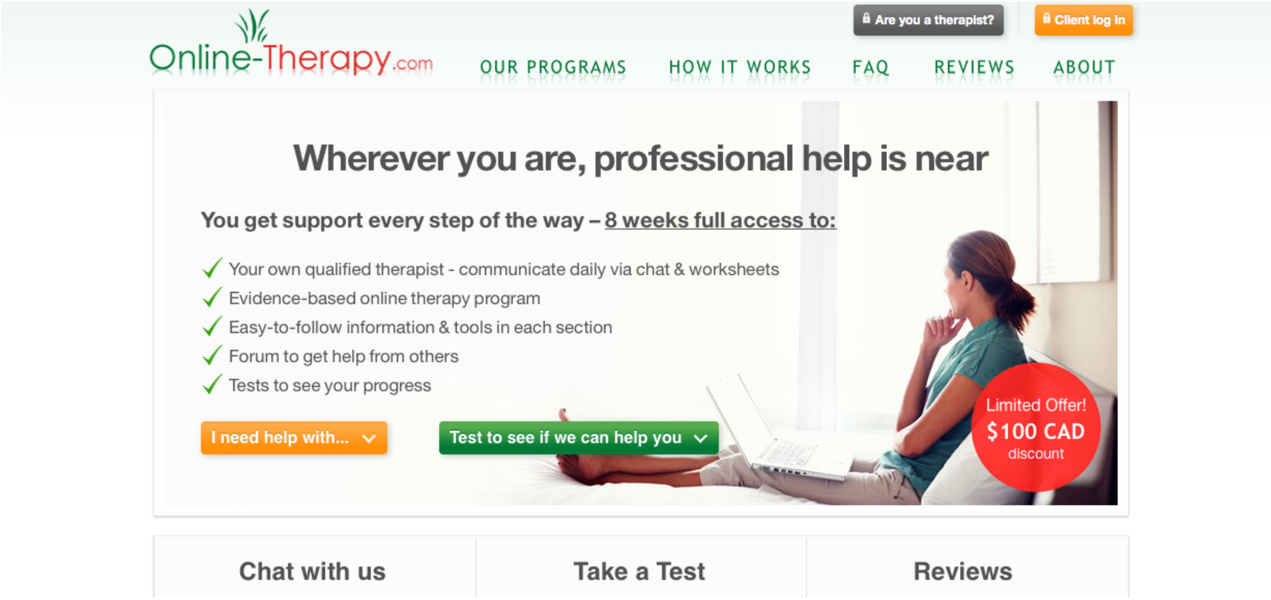

Online-Therapy welcome page.

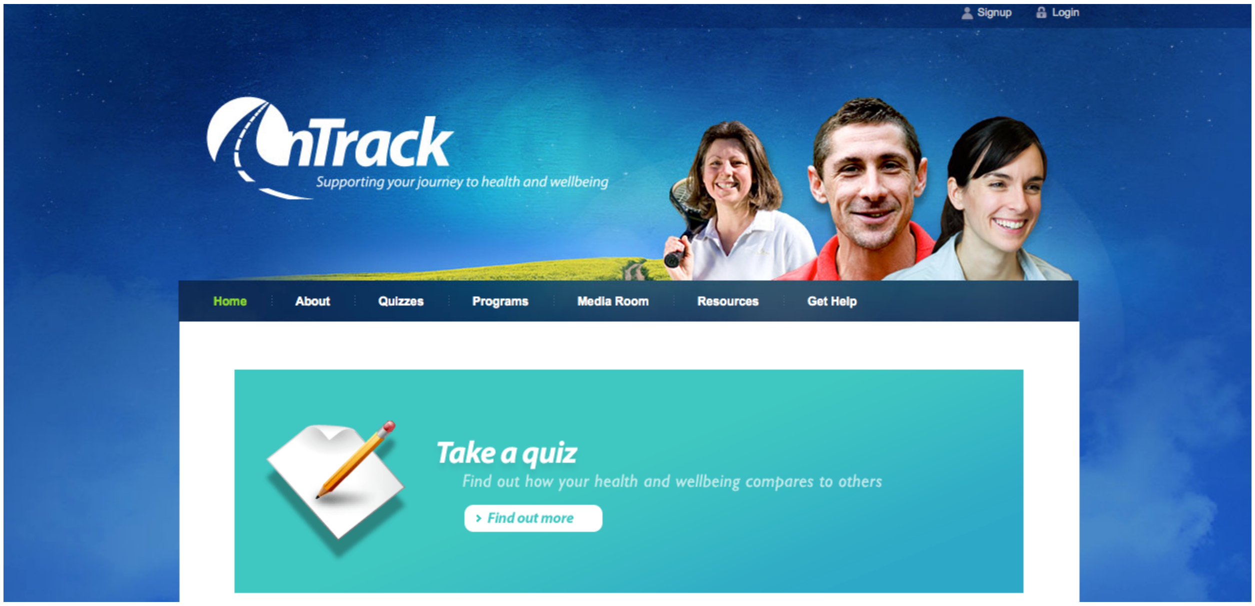

OnTrack welcome page.

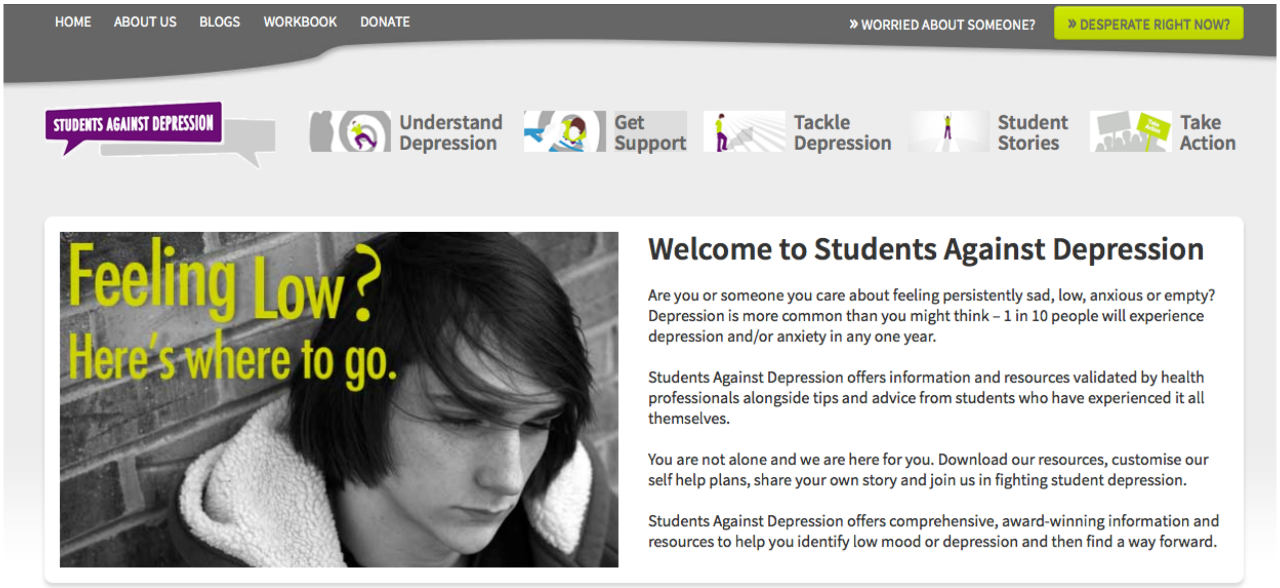

Students Against Depression welcome page.

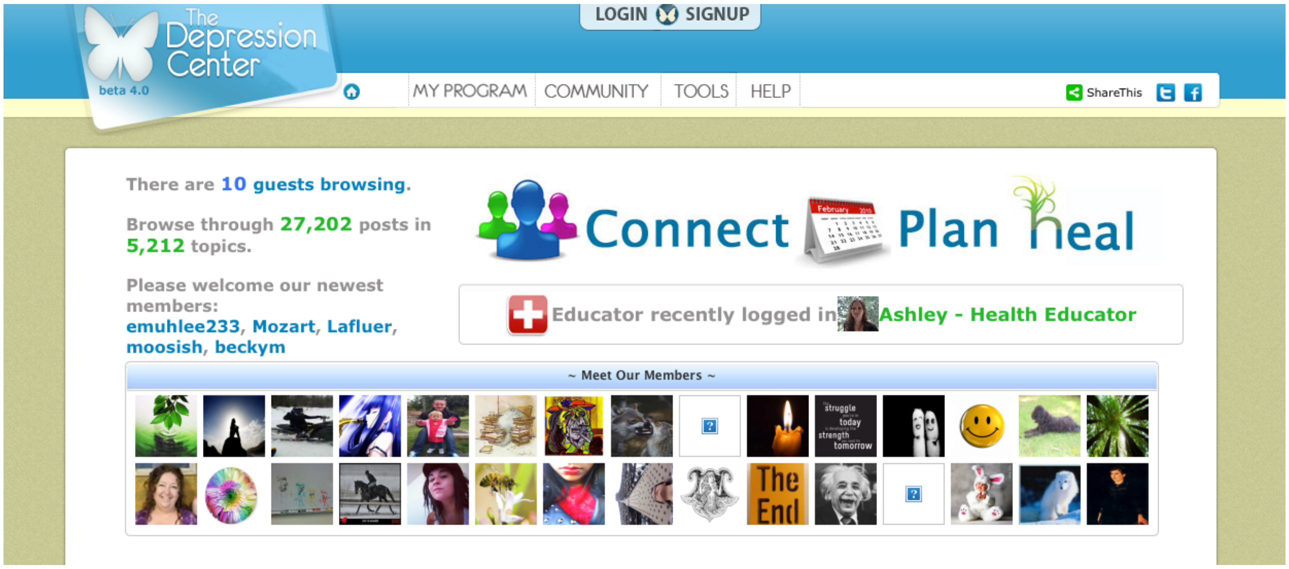

The Depression Center welcome page.

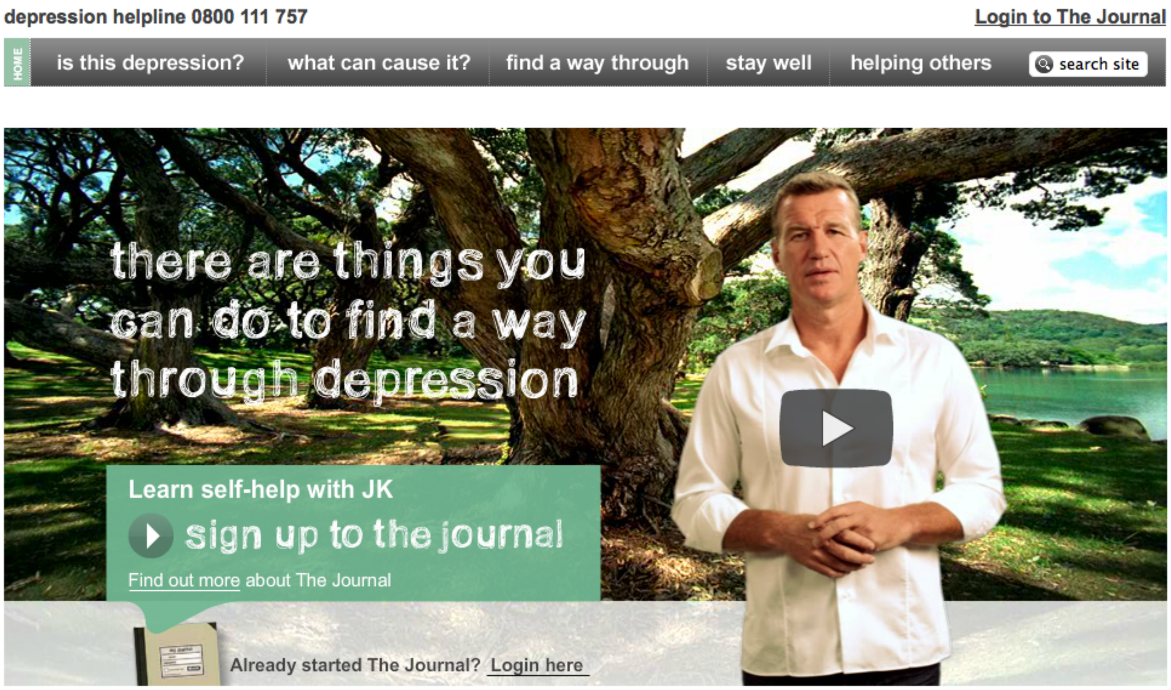

The Journal welcome page.

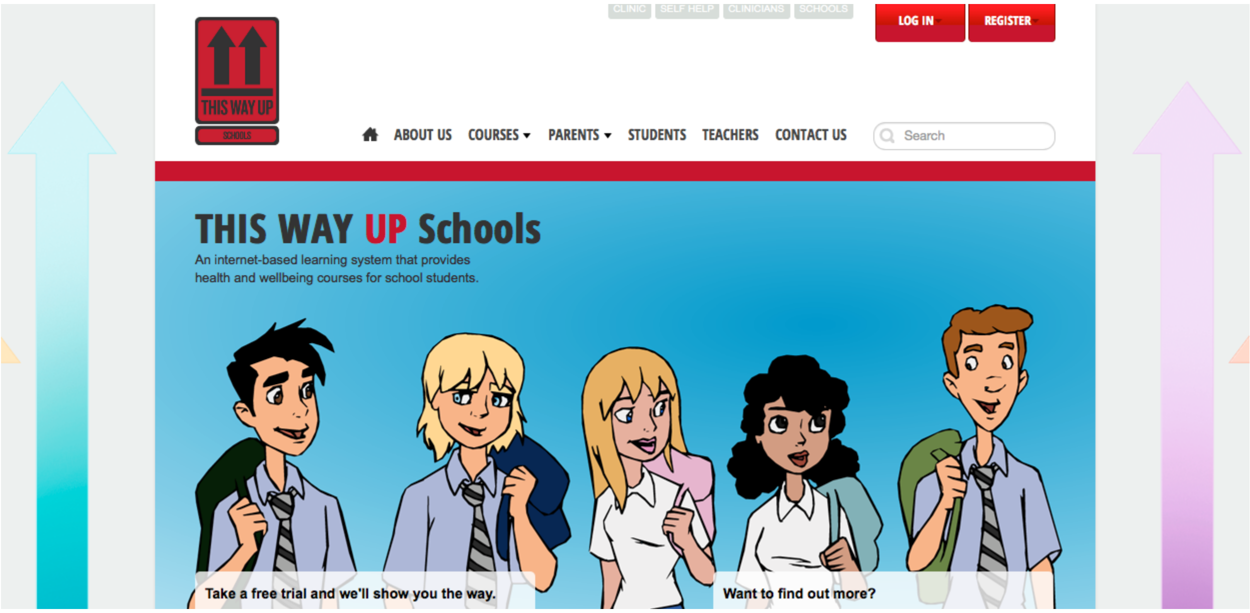

This Way Up - Schools course welcome page.
